# An ethnobotanical review of medicinal plants used for treating hemorrhoids in Thailand

**DOI:** 10.3389/fphar.2026.1705134

**Published:** 2026-04-24

**Authors:** Angkhana Inta, Rapeeporn Kantasrila, Prateep Panyadee, Akharasit Bunsongthae, Yinxian Shi, Yao Fu, Ruyu Yao

**Affiliations:** 1 Yunnan International Joint Laboratory of Health Plant Resources Development, Department of Economic Plants and Biotechnology, Yunnan Key Laboratory for Wild Plant Resources, Kunming Institute of Botany, Chinese Academy of Sciences, Kunming, China; 2 Department of Biology, Faculty of Science, Chiang Mai University, Chiang Mai, Thailand; 3 Queen Sirikit Botanic Garden (QSBG), The Botanical Garden Organisation, Chiang Mai, Thailand; 4 Department of Biology, Faculty of Science and Technology, Chiang Mai Rajabhat University, Chiang Mai, Thailand; 5 Southeast Asia Biodiversity Research Institute, Chinese Academy of Sciences, Nay Pyi Taw, Myanmar

**Keywords:** anti-inflammatory plants, piles, quantitative ethnobotany, Rid Si Duang, Thai medicine, traditional

## Abstract

Hemorrhoids is one of the most prevalent diseases affecting people’s health both in Thailand and worldwide. The therapeutic use of medicinal plants for hemorrhoidal treatments is widespread across many countries, supported by evidence demonstrating the efficacy of their pharmacological activity. This study aims to (1) conduct an in-depth literature review on plants used in Thailand for hemorrhoid treatment and (2) identify the most promising species in traditional Thai medicine to effectively treat hemorrhoids. A total of 53 references were methodically reviewed. The important medicinal plants were determined using ethnobotanical indices including use-value (UV) and choice value of species (CVs), and the important species were selected for an in-depth review of their pharmacological activities. Our literature review found that there were 181 species belonging to 147 genera and 78 families used to treat hemorrhoids among 18 ethnic groups. The most cited species were *Cissus quadrangularis*, *Biancaea sappan*, *Croton persimilis*, *Mimosa pudica*, *Aegle marmelos*, *Leea indica*, *Plumbago indica*, *Rotheca serrata*, and *Tectona grandis*. Prevalent pharmacological activities such as anti-inflammatory, anti-oxidative, anti-microbial, anti-hemorrhoid, analgesic, and vasorelaxant activities are found in key species used for hemorrhoid treatment. This study identifies the most important species for ethnopharmacological explorations in hemorrhoid treatments, which is expected to inspire the selection of medicinal plants for development into pharmaceutical products.

## 1 Introduction

Medicinal plants have long served as essential resources for treating numerous diseases across diverse cultures worldwide, forming the backbone of traditional and modern medicine alike (Kiyinlma et al., 2019; Yao et al., 2023). Notably, approximately 80% of Western pharmaceuticals are plant-derived (Hedberg, 1993). Their accessibility and affordability make medicinal plants a cornerstone of healthcare in many regions, including in the management of hemorrhoids (Soladoye et al., 2010; Yao et al., 2024). Ethnobotanical documentation of plant-based remedies provides invaluable insights, often sparking new pharmaceutical innovations grounded in traditional knowledge (Yao et al., 2021).

Hemorrhoids (piles), or Rid-Si-Duang in Thai, is among the most common gastrointestinal ailments globally (Mosavat et al., 2015b), characterized by the inflammation of venous blood vessels around the rectum and anus (Septadina and Veronica, 2015). The condition—classified into internal and external types based on the location—affects all age groups but is especially prevalent among individuals over 50 years of age (Haas et al., 1984; Bailey, 2004). More than half of the global population experiences hemorrhoids at some stage, impacting quality of life and daily functioning across varied regions including Africa, Iran, and the United States (Soladoye et al., 2010; Duke, 1989; Amiri et al., 2023; Liebach and Cerda, 1991). Contributing factors include low-fiber diets, constipation, prolonged sitting, pregnancy, genetic predispositions, and aging (Gami, 2011; Ganz, 2013).

Conventional treatments for hemorrhoids range from dietary adjustments and fluid intake to pharmaceutical and surgical interventions (Lorenzo-Rivero, 2009; Mosavat et al., 2015a; Karimi et al., 2016). However, the side effects of modern therapies, such as pain, discomfort, and postoperative complications, have driven growing interest in plant-based remedies due to their natural healing properties and relatively lower risk profiles (Priyadarshni and Arunima, 2014). Across countries—including Bulgaria, China, India, Nigeria, Tanzania, and Turkey—medicinal plants have demonstrated efficacy in improving vascular health and alleviating hemorrhoidal symptoms (MacKay, 2001; Kochmarov et al., 2015; Shi et al., 2020; Kacholi and Mvungi Amir, 2022; Erbay and Sarı, 2018). Critical phytochemicals such as flavonoids, tocotrienols, terpenoids, and alkaloids offer anti-inflammatory, antioxidant, analgesic, laxative, and anti-hemorrhoidal activities (Alonso-Coello et al., 2006; Gami, 2011; Hashempur et al., 2017; Amiri et al., 2023).

In Thailand, hemorrhoids remain a significant health concern, notably among the 45–65 age group (Lohsiriwat, 2017). Although various surgical and non-surgical options exist (Senvorasinh et al., 2019), traditional plant-based therapies are widely favored due to their efficacy and minimal side effects (Mukda and Subhadhirasakul, 2014).

Given this context, the present study aims to 1) provide an ethnobotanical review of medicinal plants used for hemorrhoid treatment in Thailand and 2) identify the ethnobotanical species most frequently and effectively utilized in traditional medicine. Findings from this research are expected to support the discovery of novel plant-based therapies for hemorrhoid management and underscore the importance of conserving Thailand’s botanical diversity.

## 2 Methods

### 2.1 Data source and search strategies

The literature review of medicinal plants used to treat hemorrhoids involved a comprehensive examination of published and unpublished research conducted between 2020 and 2023. Screening and data extraction were performed by three reviewers. Data were systematically extracted from several renowned databases and sources, including the ThaiLIS Digital Collection (https://tdc.thailis.or.th/), PubMed (Medline), Google Scholar, Web of Sciences, Scopus, Thai-Journal Citation Index, and Science Direct. Additionally, unpublished data were sourced from Ethnobotany and Northern Thai Flora Laboratory at Chiang Mai University’s Department of Biology and the library of Queen Botanic Organization. The literature review encompassed articles written in both Thai and English.

The search strategy employed a variety of key terms including “hemorrhoids AND Thailand AND ethnobotany,” “medicinal plant to treat hemorrhoids AND Thailand,” “hemorrhoid disease AND Thailand,” “gastrointestinal illnesses AND Thailand,” and “Rid-Si-Duang.” To avoid duplication of data, we selected information from these sources when data were presented in both theses and journal articles. The extracted data included the year of publication, study subjects, ethnic groups, provinces in Thailand, and the number of species used for treating hemorrhoids.

### 2.2 Screening criteria

The screening process was carried out in two phases. In the first phase, title and abstract screening of the obtained literature was done. Subsequently, the suitable articles were downloaded and accessed onsite for further critical examination. The literature screening was based on the inclusion and exclusion criteria.

### 2.3 Inclusion criteria

The published documents, in the form of articles, book chapters, reports, and theses, related to plants used for hemorrhoid treatment from 1990–2023 in Thailand were included in the study.

### 2.4 Exclusion criteria

The following types of data were strictly excluded from the study:

1. Data from review articles and experimental studies.
2. Data from studies that did not incorporate Thailand and plants used for hemorrhoid treatment.
3. Ethnomedicinal literature not encompassing information such as plant names (scientific or common) and hemorrhoid disease treatment.

A total of 1,394 articles were initially identified through key term searches in the selected databases. After removing duplicate articles, the remaining records were subjected to a two-phase screening process. In the first phase, titles and abstracts were screened to assess their relevance. Subsequently, potentially eligible articles were retrieved and examined in full text through onsite access for further critical evaluation. The screening process was conducted in accordance with the predefined inclusion and exclusion criteria. After applying these criteria, 53 relevant articles were ultimately selected and included in the final review.

### 2.5 Data retrieval

The ethnobotanical data, including scientific names and family names, were verified using Plants of the World Online (powo.science.kew.org/) and Flora of Thailand. Information on plant parts used, methods of preparation, and medicinal application was extracted from the original reports and categorized into groups according to The Economic Botany Data Collection Standard (Cook, 1995) and calculated as percentage.

### 2.6 Data analysis

For the calculation of ethnobotanical indices, the collected ethnobotanical information was systematically organized under the category of “use reports.” Each “use report” represents an instance in which a specific plant species was used to treat hemorrhoids, along with details of its preparation. In this context, a “use report” refers to the use of a particular plant part for a specific medicinal purpose by a particular ethnic group, as documented in a primary source. When a single species is reported to have multiple uses, each use is recorded as a separate use report. Any use reports lacking a scientific plant name were excluded from analysis. We carefully reviewed each use report in every reference, treating references from primary bibliographical sources as “informants” (Phumthum et al., 2018; Tardío et al., 2016). To assess the significance of medicinal plants for hemorrhoid treatment, we calculated ethnobotanical indices such as use-value (UV) and choice value of species (CVs), which provide quantitative measures of each species’ relative importance in this context. To corroborate traditional use and reported therapeutic potential, the ethnobotanical plant species were reviewed for documented pharmacological activities, including anti-inflammatory, analgesic, antioxidant, antimicrobial, anti-hemorrhoidal, vasorelaxant, local anesthetic, venotonic, and wound-healing properties.

### 2.7 Use-value (UV)

The use-value is an ethnobotanical index, which is used to find the key species used to treat a particular disease among informants. UV was calculated according to the following formula (Reyes-García et al., 2006):

$$UV=\frac{\sum Ui}{N},$$

where Ui represents the number of use reports in each species for treating hemorrhoids and N represents the total number of informants. Species with a high UV were popular to treat hemorrhoids; in contrast, low UV indicates that this species is mentioned in few reports to treat hemorrhoids.

### 2.8 Choice value of species (CVs)

This index is a tool used to define the most preferred species for treating hemorrhoids. The CVs was calculated using the following formula (Kremen et al., 1998):

$$CVs=\frac{\text{Pcs}}{\text{Sc}}\times 100,$$

where Pcs is the percentage of informants that cited the species to treat hemorrhoids and Sc is the total number of species mentioned by all informants for treating hemorrhoids.

Choice values of species range from 0 to 100; high CVs indicate that the plant species is preferred for treating hemorrhoids.

## 3 Results

### 3.1 Diversity of plants for hemorrhoid treatment

**FIGURE 1:**
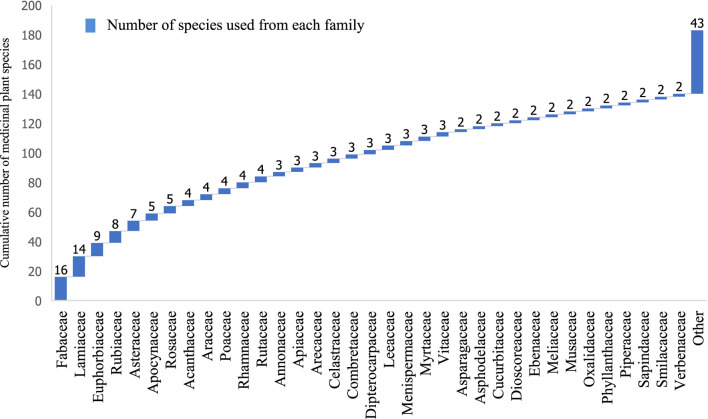
The cumulative number of medicinal plant species from each family used for treating hemorrhoids in Thailand.

In this review, traditional knowledge from 18 ethnic groups, based on 53 references covering 33 provinces, regarding the treatment of hemorrhoids was reviewed (Supplementary Table S1). Predominantly, this knowledge is derived from Tai Yuan (10 references), followed by Karen (8 references) and Tai Yai (4 references), traditions. The majority of these studies were conducted in northern Thailand, especially in Chiang Mai, Mae Hong Son, Nan, Lamphun, and Chiang Rai. In total, 288 use reports were compiled, encompassing 181 species, 147 genera, and 78 families from these 53 references (Supplementary Table S2; Figure 1). Notably, the families with the most species used in hemorrhoid treatment include Fabaceae (15 species), Lamiaceae (14 species), Euphorbiaceae (9 species), Rubiaceae (8 species), Asteraceae (7 species), Apocynaceae (6 species), and Rosaceae (5 species).

### 3.2 Plant parts used, methods of preparation, and medicinal application

**FIGURE 2:**
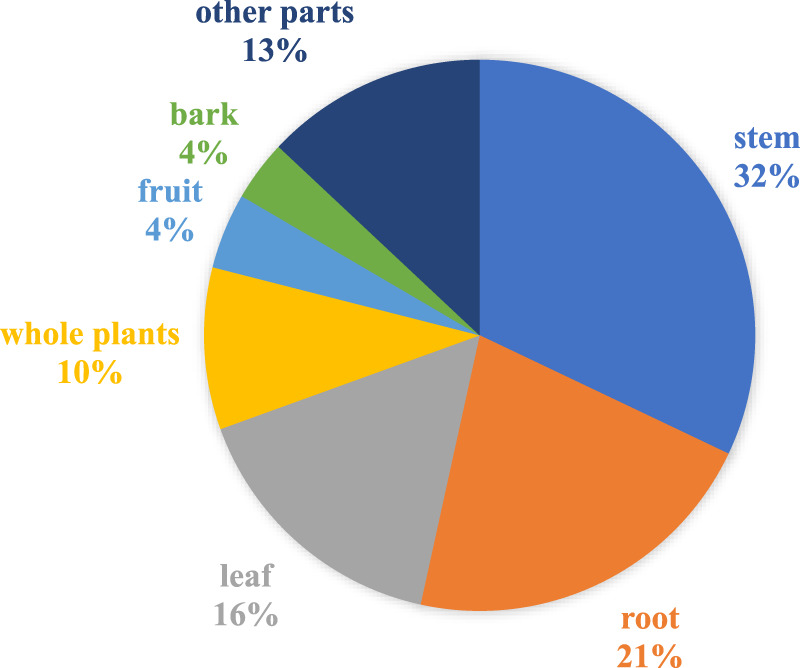
Medicinal plant parts used to treat hemorrhoids in Thailand (percentage).

In our review, the most common plant part used for treating hemorrhoids was the stem, accounting for 32% of the cases, followed by the root (21%), leaf (16%), and whole plants (10%) (Supplementary Table S2; Figure 2). Less common parts such as the bulb, corn cob, heart wood, leaf galls, seed oil, tuber, and vine each had only one use report.

**FIGURE 3:**
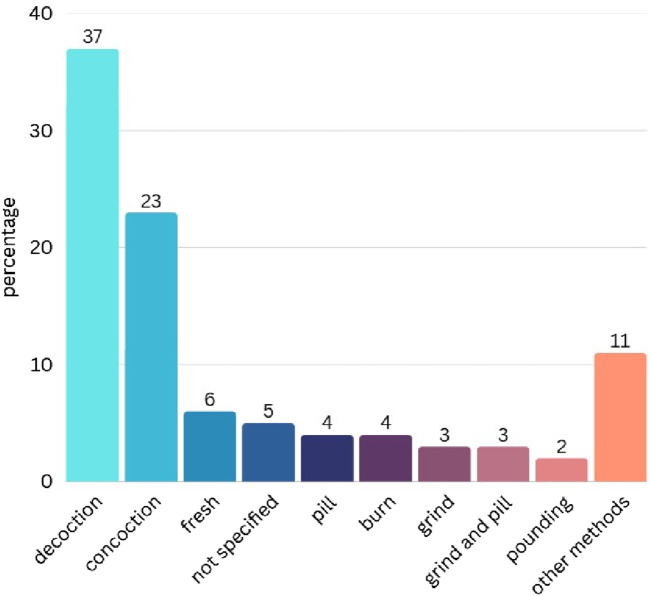
Method of preparation of medicinal plants to treat hemorrhoids in Thailand (percentage).

Regarding the usage of medicinal plants for hemorrhoid treatment, both single-drug and recipe-based approaches were observed. A notable variety of preparation methods were documented. The most prevalent method was decoction (37%), followed by concoction (23%), using fresh material (6%), pills (4%), burning (4%), grinding (3%), a combination of grinding and pill formation (3%), and pounding (2%). However, it was observed that many use reports from original references did not specify the method of preparation (Figure 3).

**FIGURE 4:**
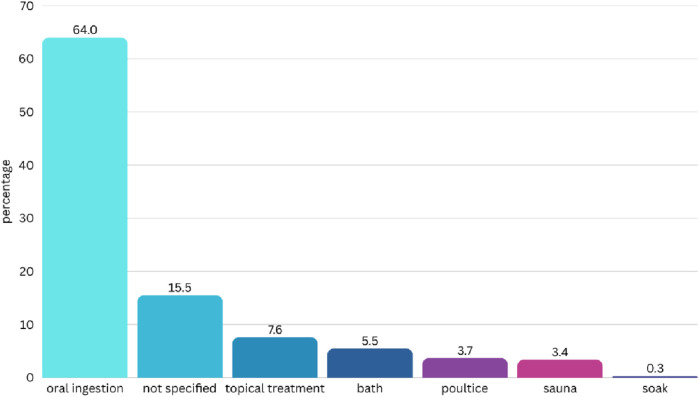
Application of medicinal plants to treat hemorrhoids in Thailand (percentage).

In case of medicinal application, oral ingestion was the most common method, representing 64% of the cases (Figure 4). Other methods included topical treatment, bath, poultices, saunas, and soaking. Similar to preparation methods, several use reports did not specify the medicinal application.

### 3.3 Ethnobotanical indices: UV and CVs

To identify the most significant ethnobotanical species for treating hemorrhoids in Thailand, UV and CVs indices were calculated. The UV, derived from use reports, ranged from 0.02 to 0.25, while the CV, based on the number of informants, ranged from 0.01 to 1.10 (Supplementary Table S2). The species with the highest UV and CV were *Cissus quadrangularis* (0.25 and 0.10), *Biancaea sappan* (0.11 and 0.06), *Croton persimilis* (0.09 and 0.04), *Mimosa pudica* (0.09 and 0.04), *Aegle marmelos* (0.08 and 0.04), *Clerodendrum chinense* (0.08 and 0.01), *Leea indica* (0.08 and 0.04), *Plumbago indica* (0.08 and 0.04), *Rotheca serrata* (0.08 and 0.04), and *Tectona grandis* (0.08 and 0.04). However, it is noteworthy that approximately 70% of the medicinal plants reviewed were reported only once. It should be emphasized that these ethnobotanical indices reflect the cultural prominence and citation frequency of medicinal plants within traditional knowledge systems rather than serving as direct measures of their therapeutic efficacy.

## 4 Discussion

### 4.1 Plant family trends in hemorrhoid treatment

This review demonstrates the widespread use of medicinal plants for hemorrhoid treatment among various ethnic groups in Thailand, particularly the Tai Yuan, to relieve symptoms such as pain, bleeding, and itching (Getachew et al., 2022). The plants identified account for approximately 8% of all medicinal plant species recorded in Thailand (Phumthum et al., 2018). Fabaceae plants are the most frequently used for hemorrhoid remedies, a trend also observed in countries such as Ethiopia, Iran, and Nigeria (Getachew et al., 2022; Hashempur et al., 2017; Soladoye et al., 2010). Fabaceae species are also widely utilized for treating a variety of other diseases (Sreekeesoon and Mahomoodally, 2014; Malik et al., 2018; Phumthum et al., 2018; Ong and Kim, 2020). Other prominent families in hemorrhoid management include Lamiaceae, Euphorbiaceae, Rubiaceae, Asteraceae, Apocynaceae, and Rosaceae. Notably, the use of Euphorbiaceae, Apocynaceae, and Asteraceae is prevalent in South-Western Nigeria for hemorrhoid management (Soladoye et al., 2010), while Lamiaceae and Asteraceae are globally recognized for their diverse medicinal uses (Michel et al., 2020). Lamiaceae, in particular, is known for its aromatic and therapeutic properties and includes more than 3,000 species.

### 4.2 Commonly used plant parts and preparation methods in hemorrhoid remedies

Stems, roots, and leaves are the most frequently used plant parts in hemorrhoid remedies, aligning with general herbal practices in Thailand (Phumthum et al., 2018). Use of leaves is especially favored in Ethiopia, Nigeria, Central Africa, and Ivory Coast for their abundance of secondary metabolites and ease of collection (Getachew et al., 2022; Ariyo et al., 2020; Ottou et al., 2020; Kiyinlma et al., 2019; Yemele et al., 2015).

Decoction is the primary preparation method due to its simplicity and effectiveness in extracting active compounds and minimizing toxicity. This method is widely adopted both in Thailand (Anderson, 1993; Phumthum et al., 2018; Kantasrila, 2021) and internationally (Coe and Anderson, 1996; Liu et al., 2009; Simbo, 2010; Ong and Kim, 2014; Malik et al., 2018), particularly for hemorrhoid treatment (Kiyinlma et al., 2019).

Decoctions were most commonly administered orally, supporting systemic therapeutic effects (Hashempur et al., 2017; Kebebew, 2016). Topical application was also reported, especially for external hemorrhoids, to help reduce inflammation, bleeding, and swelling (Getachew et al., 2022; Kacholi and Mvungi Amir, 2022). Nevertheless, many studies lacked details on the mode of application, highlighting the need for more comprehensive ethnobotanical documentation.

### 4.3 Significant medicinal plants for hemorrhoid treatments

The most significant medicinal plants for hemorrhoid treatment were identified using UV and CV, with high values suggesting promising efficacy and popularity. To confirm traditional use and efficacy, these plants were reviewed for relevant pharmacological activities, including anti-inflammatory, analgesic, antioxidant, antimicrobial, anti-hemorrhoid, vasorelaxant, local anesthetic, venotonic, and wound-healing effects (Lohsiriwat, 2012; Rahimi and Abdollahi, 2013; Astana et al., 2011). Key bioactive compounds associated with anti-hemorrhoidal activity include flavonoids, phenolic compounds, terpenoids, steroids, saponins, alkaloids, triterpenes, and tannins (Rahimi and Abdollahi, 2013). Notably, flavonoids, tannins, and terpenoids are linked with reducing symptoms such as bleeding, pain, and inflammation (Alonso-Coello et al., 2006). Prominent species identified in this study were *C. quadrangularis, B. sappan, C. persimilis, M. pudica, A. marmelos, C. chinense, L. indica, P. indica, R. serrata,* and *T. grandis* (Supplementary Table S3).

*Cissus quadrangularis* had the highest UV and CV, being cited in 10 sources. In Thailand, it is recognized for vasoconstrictive and anti-inflammatory effects (Panthong et al., 2007; Bhujade et al., 2012). In India, a mixture of *C. quadrangularis* stem and honey is used to ease pain and inflammation (Bhujade et al., 2012), and it has been reported in hemorrhoid treatment (Rahimi and Abdollahi, 2013). The methanol extract inhibits local mediators and nociceptors involved in pain and vascular contraction (Panthong et al., 2007). The active phytochemicals include flavonoids, triterpenoids, and stilbene derivatives (Panthong et al., 2007; Rahimi and Abdollahi, 2013).

*Biancaea sappan*, widely used in Thai folk medicine, treats ailments such as tuberculosis, diarrhea, dysentery, and anemia (Sireeratawong et al., 2011). In Chinese medicine, it is valued for its analgesic and anti-inflammatory properties, as well as for increasing blood circulation and stimulating menstruation (Commission, 2010). Important compounds include alkaloids, steroids, phenols, flavonoids, terpenoids, triterpenoids, xanthone, coumarin, tannins, and terpenoids (Thangal et al., 2022; Vij et al., 2023), which support antioxidant (Suwan et al., 2018), anti-inflammatory (Tewtrakul et al., 2015), vasorelaxant (Yan et al., 2015), and antibacterial (Vij et al., 2023) properties.

*Croton persimilis* is used by Thai Yuan, Karen, and Lahu communities for hemorrhoid management, as well as for cough, pain, muscle pain, and dizziness (Appamaraka et al., 2023; Pongamornkul, 2017). In Myanmar, it is used to treat hemorrhoids, flatulence, bowel issues, blood clotting, dysentery, and boils (DeFilipps and Krupnick, 2018). Its phytochemicals include alkaloids, flavonoids, saponins, tannins, terpenoids, steroids, phenolic compounds, and coumarin (Rattanapunya et al., 2021), supporting antioxidant (Rattanapunya et al., 2021) and anti-inflammatory benefits (DeFilipps and Krupnick, 2018).

*Mimosa pudica* is used internally and externally by the Tai Yai, Thai Yuan, and Karen groups in Thailand and is consumed with milk for hemorrhoid management in Myanmar (DeFilipps and Krupnick, 2018). In Thailand, it is used to treat a range of disorders, including urological, endocrine, digestive, musculoskeletal, and cardiovascular diseases (Panyadee, 2022). It is also used in Chinese medicine for treating tuberculosis, insomnia, anxiety, and trauma (Xu et al., 2019). Key phytochemicals are alkaloids, flavonoids, saponins, triterpenes, glycosides, quinines, tannins, and coumarin (Gandhiraja et al., 2009; Racadio, 2016), yielding anti-inflammatory (Patel and Bhutani, 2014), antioxidant (Kokane et al., 2009; Zhang et al., 2011), wound-healing (Kokane et al., 2009; Lakshmibai and Amirtham, 2018), and analgesic (Patro et al., 2015) activities.

*Aegle marmelos* was historically used for hemorrhoid management in Persia and Iran (Hashempur et al., 2017) and for addressing bowel and diarrhea issues in Myanmar (DeFilipps and Krupnick, 2018). The leaf and root flavonoids show anti-nociceptive effects (Choudhary et al., 2017), while flower extracts have anti-inflammatory effects in rats (Kumari et al., 2014), and leaf extracts demonstrate analgesic and antipyretic properties (Arul et al., 2005).

*Leea indica* roots and stems are used for treating hemorrhoids, and its shoots help reduce swelling (Raghavendra, 2018; Singh et al., 2019). It is also applied to ease joint pain, sores, leprosy, eczema, fractures, allergies, and diarrhea (Hossain et al., 2021). Main compounds include alkaloids, coumarin, flavonoids, saponins, phenolics, terpenoids, phthalic acid derivatives, and steroids (Saha et al., 2007; Srinivasan et al., 2008). It displays antioxidant (Wong et al., 2012), analgesic (Emran et al., 2012), anti-inflammatory (Sakib et al., 2021), and wound-healing (Azizi et al., 2016) effects.

*Plumbago indica* is mainly used internally for hemorrhoid management (Khatun, 2023) and also for bronchitis, digestive disorders, neurological conditions, and inflammatory diseases (Priyanjani et al., 2021). Active compounds are alkaloids, flavonoids, steroids, and tannin (Eldhose et al., 2013). Methanolic extracts reduce pain (Paul and Saha, 2012) and exert anti-inflammatory activity through naphthoquinone plumbagin (Checker et al., 2009). Root extracts are rich in antioxidant compounds (Eldhose et al., 2013). *Plumbago zeylanica*, from the same genus, is also reported for hemorrhoid treatment (Giday et al., 2007; Jima and Megersa, 2018).

*Rotheca serrata* has a long history in Indian (Patel et al., 2014), Myanmar (DeFilipps and Krupnick, 2018), and Thai traditional medicine (Pongamornkul, 2017) for management of pain, inflammation, rheumatism, and respiratory disorders. Its key compounds are phenolics, tannins, flavonoids, and saponins (Vidya et al., 2007; Patel et al., 2014), and it exhibits hepatoprotective, antioxidant, and anti-inflammatory activities (Patel et al., 2014).

*Tectona grandis* is known for its wound-healing properties (Majumdar, 2005) and is used in Thai medicine for diabetes, blood pressure, stomach ache, and pain (Pongamornkul, 2017). It demonstrates potent analgesic (Giri and Varma, 2015), anti-inflammatory (Giri and Varma, 2015; Han et al., 2023), antioxidant (Han et al., 2023), and wound-healing effects (Varma and Giri, 2013). Major phytochemicals include triterpenoids, steroids, lignans, fatty esters, phenolics, flavonoids, saponins, alkaloids, and tannins (Vyas et al., 2019).

In summary, these plants, notable for their UV and CV, are used not only traditionally for hemorrhoid management but also for treating numerous other ailments in different regions, with substantial pharmacological evidence to support their effects. To validate their medicinal use, further clinical trials are recommended.

## 5 Conclusion

Hemorrhoids is one of the most prevalent diseases in Thailand. Various ethnic groups use medicinal plants to treat this condition. Some plant species, such as *C. quadrangularis*, are commonly used for hemorrhoid treatments across multiple ethnic groups. This study identifies promising plant species for the treatment of hemorrhoids in Thailand, based on their high ethnobotanical index and reported pharmacological activities. These pharmacological properties support the effectiveness of medicinal plants in treating hemorrhoids. All promising species exhibit various pharmacological activities, including antioxidant, anti-inflammatory, analgesic, antimicrobial, anti-hemorrhoid, vasorelaxant, and venotonic effects, along with local anesthetic and wound-healing properties. These activities are directly related to the treatment of hemorrhoids.

This literature review can serve as a foundation for selecting plants for future clinical studies on hemorrhoid treatments and for developing medicinal plants into commercial products. Moreover, it provides valuable information as an alternative treatment for hemorrhoids, particularly considering the side effects associated with surgical hemorrhoidectomy. Based on quantitative ethnobotany, the present work selects the most important medicinal plants used for hemorrhoid treatments in Thailand. Further studies should focus on the pharmacological validation and clinical testing of promising plant species identified through ethnobotanical surveys. Although long-standing traditional use reflects cultural importance, it does not necessarily guarantee safety or clinical efficacy. Investigating the mechanisms of action, safety profiles, and active compounds of these plants will help bridge the gap between traditional knowledge and scientific evidence. In addition, exploring the potential of lesser-known or novel plant species may lead to the discovery of alternative therapies for hemorrhoid treatment. Continued documentation of traditional practices is also essential to preserve ethnobotanical knowledge for future generations.

However, it is important to note the limitations of the current literature in Thailand. Many existing studies rely primarily on ethnobotanical surveys based on self-reported or recalled data, with limited pharmacological or clinical validation. There is a lack of standardized research methodologies, and much of the relevant research is published in Thai-language journals, which restricts accessibility and recognition in the international scientific community. Addressing these limitations through well-designed pharmacological studies and clinical trials will be crucial for fully validating and utilizing Thai medicinal plant resources for hemorrhoid treatment.

## Data Availability

The original contributions presented in the study are included in the article/Supplementary Material; further inquiries can be directed to the corresponding authors.

## References

[B1] Alonso-Coello P. Zhou Q. Martinez-Zapata M. Mills E. Heels-Ansdell D. Johanson J. (2006). Meta-analysis of flavonoids for the treatment of haemorrhoids. Br. J. Surg. 93 (8), 909–920. 10.1002/bjs.5378 16736537

[B2] Amiri M. M. Garnida Y. Almulla A. F. Abduljabbar A. S. Jalil A. T. Mazaheri Y. (2023). Herbal therapy for hemorrhoids: an overview of medicinal Plants Affecting hemorrhoids. Adv. Life Sci. 10 (1), 22–28. 10.62940/als.v10i1.1513

[B3] Anderson E. F. (1993). Plant and people of the golden triangle: ethnobotany of the hill tribe of the Northern Thailand. Portland, Oregon: Whitman College and Desert Botanical Garden.

[B4] Appamaraka S. Saensouk S. Saensouk P. Junsonguang A. Setyawan A. D. (2023). Ethnobotanical knowledge of medicinal plants in the Don Pu Ta Forest by Kaloeng Ethnic Group, Sakon Nakhon Province, northeastern Thailand. Biodiversitas 24 (8), 4617–4635. 10.13057/biodiv/d240844

[B5] Ariyo O. Usman M. Emeghara U. Olorukooba M. Fadele O. Danbaki C. (2020). Indigenous curative plants used in the treatment of piles from Akinyele local government area, Ibadan, Oyo State, Nigeria. Annu. Res. Rev. Biol. 35 (6), 78–89. 10.9734/arrb/2020/v35i630238

[B6] Arul V. Miyazaki S. Dhananjayan R. (2005). Studies on the anti-inflammatory, antipyretic and analgesic properties of the leaves of *Aegle marmelos* Corr. J. Ethnopharmacol. 96 (1–2), 159–163. 10.1016/j.jep.2004.09.013 15588665

[B7] Astana P. Nisa U. Triyono A. Ardiyanto D. Fitriani U. Zulkarnain Z. (2011). “Medicinal plants used by traditional healers for hemorrhoid treatment in Borneo island: ethnopharmacological study RISTOJA,” in IOP conference series: earth and environmental science (Vancouver, Canada: IOP Publishing).

[B8] Azizi W. Sunzida N. Azad A. (2016). The screening of local herbs in treating non healing wounds and diabetic foot ulcers complications using nih 3t3 mouse fibroblast and raw 264.7 mouse macrophage cells. Pharmacol. 4, 139–145.

[B9] Bailey H. (2004). Innovations for an age-old problem: hemorrhoids in the female patient. Female Patient 29 (1), 17–23.

[B10] Bhujade A. M. Talmale S. Kumar N. Gupta G. Reddanna P. Das S. K. (2012). Evaluation of *Cissus quadrangularis* extracts as an inhibitor of COX, 5-LOX, and proinflammatory mediators. J. Ethnopharmacol. 141 (3), 989–996. 10.1016/j.jep.2012.03.044 22484053

[B12] Checker R. Sharma D. Sandur S. K. Khanam S. Poduval T. (2009). Anti-inflammatory effects of plumbagin are mediated by inhibition of NF-kappaB activation in lymphocytes. Int. Immunopharmacol. 9 (7–8), 949–958. 10.1016/j.intimp.2009.03.022 19374955

[B13] Choudhary Y. Saxena A. Kumar Y. Kumar S. Pratap V. (2017). Phytochemistry, pharmacological and traditional uses of *Aegle marmelos* . Pharm. Biosci. J. 5 (5), 27–33. 10.20510/ukjpb/5/i5/166553

[B14] Coe F. G. Anderson G. J. (1996). Ethnobotany of the Garifuna of eastern Nicaragua. Econ. Bot. 50 (1), 71–107. 10.1007/bf02862114

[B15] Commission C. P. (2010). The Chinese pharmacopoeia. Beijing: China Medical Science Press.

[B101] Cook F. E. M. (1995). Economic botany data collection standard. Royal Botanic Gardens, Kew.

[B16] DeFilipps R. A. Krupnick G. A. (2018). The medicinal plants of Myanmar. PhytoKeys 102, 1–341. 10.3897/phytokeys.102.24380 30002597 PMC6033956

[B17] Duke J. (1989). Foods as pharmaceuticals. Herbs. 89, 22–25.

[B18] Eldhose B. Notario V. Latha M. (2013). Evaluation of phytochemical constituents and *in vitro* antioxidant activities of *Plumbago indica* root extracts. J. Pharmacogn. Phytochem. 2 (4), 157–161.

[B19] Emran T. B. Rahman M. A. Hosen S. Z. Rahman M. M. Islam A. M. T. Chowdhury M. A. U. (2012). Analgesic activity of Leea indica. burm. f. merr. Phytopharm. 3 (1), 150–157.

[B20] Erbay M. Ş. Sarı A. (2018). Plants used in traditional treatment against hemorrhoids in Turkey. Marmara Pharm. J. 22 (2), 110–132. 10.12991/mpj.2018.49

[B21] Gami B. (2011). Hemorrhoids–a common ailment among adults, causes and treatment: a review. Int. J. Pharm. Pharm. Sci. 3 (Suppl. 5), 5–13.

[B22] Gandhiraja N. Sriram S. Meenaa V. Srilakshmi J. K. Sasikumar C. Rajeswari R. (2009). Phytochemical screening and antimicrobial activity of the plant extracts of *Mimosa pudica* L. against selected microbes. Ethnobot. Leafl. 2009 (5), 8.

[B23] Ganz R. A. (2013). The evaluation and treatment of hemorrhoids: a guide for the gastroenterologist. Clin. Gastroenterol. Hepatol. 11 (6), 593–603. 10.1016/j.cgh.2012.12.020 23333220

[B24] Getachew M. Belayneh A. Kebede B. Alimaw Y. Biyazin Y. Abebaw A. (2022). Medicinal plants used for management of hemorrhoids in Ethiopia: a systematic review. Heliyon 8 (8), e10211. 10.1016/j.heliyon.2022.e10211 36033288 PMC9403375

[B25] Giday M. Teklehaymanot T. Animut A. Mekonnen Y. (2007). Medicinal plants of the Shinasha, Agew-awi and Amhara peoples in northwest Ethiopia. J. Ethnopharmacol. 110 (3), 516–525. 10.1016/j.jep.2006.10.011 17101251

[B26] Giri S. P. Varma S. B. (2015). Analgesic and anti-inflammatory activity of *Tectona grandis* Linn. Stem Extract. J. Basic Clin. Physiol. Pharmacol. 26 (5), 479–484. 10.1515/jbcpp-2014-0043 25901713

[B27] Haas P. A. Fox T. A. Haas G. P. (1984). The pathogenesis of hemorrhoids. Dis. Colon Rectum 27, 442–450. 10.1007/BF02555533 6745015

[B28] Han M. Yang F. Zhang K. Ni J. Zhao X. Chen X. (2023). Antioxidant, anti-inflammatory and anti-diabetic activities of *Tectona grandis* methanolic extracts, fractions, and isolated compounds. Antioxidants 12 (3), 664. 10.3390/antiox12030664 36978912 PMC10044725

[B29] Hashempur M. H. Khademi F. Rahmanifard M. Zarshenas M. M. (2017). An evidence-based study on medicinal plants for hemorrhoids in Medieval Persia. J. Evid. Based Complement. Altern. Med. 22 (4), 969–981. 10.1177/2156587216688597 29228790 PMC5871264

[B30] Hedberg J. M. (1993). The role of exercise twining in the treatment of haemorrhoids. Mcd 30, 193–206.

[B31] Hossain F. Mostofa M. G. Alam A. K. (2021). Traditional uses and pharmacological activities of the genus leea and its phytochemicals: a review. Heliyon 7 (2), e06222. 10.1016/j.heliyon.2021.e06222 33659746 PMC7892933

[B32] Jima T. T. Megersa M. (2018). Ethnobotanical study of medicinal plants used to treat human diseases in Berbere District, Bale Zone of Oromia Regional State, South East Ethiopia. Evid. Based Complement. Altern. Med. 2018, 8602945. 10.1155/2018/8602945 30105073 PMC6076952

[B33] Kacholi D. S. Mvungi Amir H. (2022). Herbal remedies used by traditional healers to treat haemorrhoids in Tabora region, Tanzania. Pharm. Biol. 60 (1), 2182–2188. 10.1080/13880209.2022.2136204 36307997 PMC9629089

[B34] Kantasrila R. (2021). *Ethnobotanical Study of medicinal plants for treating musculoskeletal disorders among skaw Karen in Chiang Mai, Thailand.* Doctor of philosophy (biodiversity and ethnobiology). Chiang Mai, Thailand: Chiang Mai University.

[B35] Karimi M. Mardani M. Parsaei P. (2016). An overview of the effectiveness of the most important native medicinal plants of Iran on hemorrhoid based on iranian traditional medicine textbooks. J. Glob. Pharma Technol. 8, 24–26. 10.56499/jppres19.612_7.3.156

[B36] Kebebew M. (2016). Knowledge of medicinal plants used in and around Fincha'a Town, Western Ethiopia. J. Pharmacogn. Phytochem. 5 (6), 110–114.

[B37] Khatun A. (2023). *Plumbago indica* L.: a review of its medicinal uses, phytochemistry, pharmacology, and toxicology. Int. J. Herb. Med. 11 (4), 31–37. 10.22271/flora.2023.v11.i4a.877

[B38] Kiyinlma C. Théodore E. D. Bernadine O. B. A. M. Yao K. Noël Z. G. (2019). Anti-Hemorrhoidal medicinal plants of the department of Issia: inventory and cytotoxicity on HFF cells of the ethanolic extract 70% of *Landolphia utilis* A. Chev.(Apocynaceae). J. Biosci. Med. 7 (11), 101–110. 10.4236/jbm.2019.711009

[B39] Kochmarov V. Kozuharova E. Naychov Z. Momekov G. Mincheva I. (2015). Ethnobotany and ethnopharmacology of *Arum maculatum* L.(Araceae) in Bulgaria with an emphasis on its effect against haemorrhoids. Int. J. Pharm. Chem. Biol. Sci. 5 (2), 394–402.

[B40] Kokane D. D. More R. Y. Kale M. B. Nehete M. N. Mehendale P. C. Gadgoli C. H. (2009). Evaluation of wound healing activity of root of *Mimosa pudica* . J. Ethnopharmacol. 124 (2), 311–315. 10.1016/j.jep.2009.04.038 19397984

[B41] Kremen C. Raymond I. Lance K. (1998). An interdisciplinary tool for monitoring conservation impacts in Madagascar. Conserv. Biol. 12 (3), 549–563. 10.1111/j.1523-1739.1998.96374.x

[B42] Kumari K. Weerakoon T. Handunnetti S. Samarasinghe K. Suresh T. (2014). Anti-inflammatory activity of dried flower extracts of *Aegle marmelos* in Wistar rats. J. Ethnopharmacol. 151 (3), 1202–1208. 10.1016/j.jep.2013.12.043 24389030

[B43] Lakshmibai R. Amirtham D. (2018). Evaluation of free radical scavenging activity of *Mimosa pudica* thorns. Asian J. Pharm. Clin. Res. 11 (11), 153–156. 10.22159/ajpcr.2018.v11i11.27426

[B44] Liebach J. Cerda J. (1991). Hemorrhoids: modern treatment methods. Hosp. Med. 53, 68.

[B45] Liu Y. Dao Z. Yang C. Liu Y. Long C. (2009). Medicinal plants used by tibetans in Shangri-la, Yunnan, China. J. Ethnobiol. Ethnomed. 5 (1), 15. 10.1186/1746-4269-5-15 19416515 PMC2684741

[B46] Lohsiriwat V. (2012). Hemorrhoids: from basic pathophysiology to clinical management. World J. Gastroenterol. 18 (17), 2009–2017. 10.3748/wjg.v18.i17.2009 22563187 PMC3342598

[B47] Lohsiriwat V. (2017). Common anorectal diseases. Bangkok: N P Press. 10.1007/s10151-013-1091-y 24178950

[B48] Lorenzo-Rivero S. (2009). Article commentary: hemorrhoids: diagnosis and current management. Am. Surg. 75 (8), 635–642. 10.1177/000313480907500801 19725283

[B49] MacKay D. (2001). Hemorrhoids and varicose veins: a review of treatment options. Altern. Med. Rev. 6 (2), 126. 11302778

[B50] Majumdar M. (2005). Evaluation of Tectona grandis leaves for wound healing activity. Bengaluru, Karnataka, India: Rajiv Gandhi University of Health Sciences. 17416566

[B51] Malik K. Ahmad M. Zhang G. Rashid N. Zafar M. Sultana S. (2018). Traditional plant based medicines used to treat musculoskeletal disorders in northern Pakistan. Eur. J. Integr. Med. 19, 17–64. 10.1016/j.eujim.2018.02.003

[B52] Michel J. Abd Rani N. Z. Husain K. (2020). A review on the potential use of medicinal plants from asteraceae and lamiaceae plant family in cardiovascular diseases. Front. Pharmacol. 11, 852. 10.3389/fphar.2020.00852 32581807 PMC7291392

[B53] Mosavat S. H. Ghahramani L. Sobhani Z. Haghighi E. R. Chaijan M. R. Heydari M. (2015a). The effect of leek (*Allium iranicum* (wendelbo)) leaves extract cream on hemorrhoid patients: a double blind randomized controlled clinical trial. Eur. J. Integr. Med. 7 (6), 669–673. 10.1016/j.eujim.2015.08.008

[B54] Mosavat S. H. Ghahramani L. Sobhani Z. Haghighi E. R. Heydari M. (2015b). Topical *Allium ampeloprasum* subsp *iranicum* (leek) extract cream in patients with symptomatic hemorrhoids: a pilot randomized and controlled clinical trial. J. Evid. Based Complement. Altern. Med. 20 (2), 132–136. 10.1177/2156587214567954 25608984

[B55] Mukda S. Subhadhirasakul S. (2014). Herbal medicines and hemorrhoid disease. JTT Med. Res. 12 (2), 122–132.

[B56] Ong H. G. Kim Y.-D. (2014). Quantitative ethnobotanical study of the medicinal plants used by the Ati negrito Indigenous group in Guimaras island, Philippines. J. Ethnopharmacol. 157, 228–242. 10.1016/j.jep.2014.09.015 25240586

[B57] Ong H. G. Kim Y.-D. (2020). Medicinal plants for gastrointestinal diseases among the kuki-chin ethnolinguistic groups across Bangladesh, India, and Myanmar: a comparative and network analysis study. J. Ethnopharmacol. 251, 112415. 10.1016/j.jep.2019.112415 31917280

[B58] Ottou P. B. M. Biyon J. B. N. Mokake S. E. Bissemb P. O. Fouda L. R. O. Foze T. N. (2020). Knowledge of tradi-practitioners on hemorrhoidal disease and anti-hemmoroidal plants in the southeast region of Cameroon: pharmacology and preliminary phytochemistry. Saudi J. Med. Pharm. Sci. 6, 321–333. 10.36348/sjmps.2020.v06i04.001

[B59] Panthong A. Supraditaporn W. Kanjanapothi D. Taesotikul T. Reutrakul V. (2007). Analgesic, anti-inflammatory and venotonic effects of *Cissus quadrangularis* linn. J. Ethnopharmacol. 110 (2), 264–270. 10.1016/j.jep.2006.09.018 17095173

[B60] Panyadee P. (2022). A review on the ethnobotany of exotic species in Thailand I: *mimosa pudica* L.(Leguminosae). Thai J. Bot. 14, 2.

[B61] Patel N. K. Bhutani K. K. (2014). Suppressive effects of *Mimosa pudica* (L.) constituents on the production of LPS-Induced pro-inflammatory mediators. EXCLI J. 29 (13), 1011–1021. 10.17877/DE290R-6920 26417317 PMC4464187

[B62] Patel J. J. Acharya S. R. Acharya N. S. (2014). *Clerodendrum serratum* (L.) Moon.–A review on traditional uses, phytochemistry and pharmacological activities. J. Ethnopharmacol. 154 (2), 268–285. 10.1016/j.jep.2014.03.071 24727551

[B63] Patro G. Bhattamisra S. K. Mohanty B. K. (2015). Analgesic, antiepileptic, and behavioral study of *Mimosa pudica* (linn.) on experimental rodents. Int. J. Nutr. Pharmacol. Neurol. Dis. 5 (4), 144–150. 10.4103/2231-0738.167502

[B64] Paul S. Saha D. (2012). Analgesic activity of methanol extract of *Plumbago indica* (L.) by acetic acid induced writhing method. Asian J. Pharm. Technol. 2 (2), 74–76. 10.33192/smb.v16i2.260949

[B65] Phumthum M. Srithi K. Inta A. Junsongduang A. Tangjitman K. Pongamornkul W. (2018). Ethnomedicinal plant diversity in Thailand. J. Ethnopharmacol. 214, 90–98. 10.1016/j.jep.2017.12.003 29241674

[B66] Pongamornkul W. (2017). “Northern Thailand ethnobotanical index,” in Thailand: Wanida karnpim limited partmership.

[B67] Priyadarshni M. Arunima S. (2014). Ethnobotanical survey of traditional medicines for hemorrhoid treatment. Int. J. Adv. Agric. Sci. Technol. 2 (2), 74–84.

[B100] Priyanjani H. Senarath R. Senarath W. Munasinghe M. (2021). Propagation, phytochemistry and pharmacology of plumbago indica-a review. J. Pharm. Res. Int. 33 (42B), 188–202.

[B68] Racadio S. P. (2016). The medicinal prospects of makahiya (*Mimosa pudica* linn) plant. Adv. Life Sci. 6, 7–12. 10.5923/J.ALS.20160601.02

[B69] Raghavendra H. (2018). Traditional uses, chemistry and pharmacological activities of *Leea indica* (Burm. f.) Merr.(Vitaceae): a comprehensive review. Int. J. Green Pharm. 12 (01). 10.22377/ijgp.v12i01.1602

[B70] Rahimi R. Abdollahi M. (2013). Evidence-based review of medicinal plants used for the treatment of hemorrhoids. Int. J. Pharmacol. 9 (1), 1–11. 10.3923/ijp.2013.1.11

[B71] Rattanapunya S. Sumsakul W. Bunsongthae A. Jaitia S. (2021). *In vitro* antioxidants and anticancer activity of crude extract isolates from euphorbiaceae in northern Thailand. Thai J. Pharm. Sci. 45 (5), 394–399. 10.56808/3027-7922.2518

[B72] Reyes-García V. Huanca T. Vadez V. Leonard W. Wilkie D. (2006). Cultural, practical, and economic value of wild plants: a quantitative study in the Bolivian amazon. Econ. Bot. 60 (1), 62–74. 10.1663/0013-0001(2006)60[62:cpaevo]2.0.co;2

[B73] Saha K. Shaari K. Lajis N. (2007). Phytochemical study on *Leea indica* (burm. F.). Merr.(Leeaceae). J. Bangladesh Chem. Soc. 20, 139–147.

[B74] Sakib S. A. Tareq A. M. Islam A. Rakib A. Islam M. N. Uddin M. A. (2021). Anti-inflammatory, thrombolytic and hair-growth promoting activity of the n-hexane fraction of the methanol extract of *Leea indica* leaves. Plants 10 (6), 1081. 10.3390/plants10061081 34072236 PMC8229947

[B75] Senvorasinh K. Phunikhom K. Sattayasai J. (2019). Anti-Hemorrhoidal activity of *Pluchea indica* leaves aqueous extract in croton oil-induced hemorrhoids in experimental animals. Srinagarind Medl. J. 34 (6), 590–594.

[B76] Septadina I. S. Veronica F. (2015). Gambaran histopatologi epitel transisional kolorektal pada pasien hemoroid. J. Kedokt. Kesehat. 2 (1), 85–91. 10.32539/jkk.v2i1.13

[B77] Shi S. Y. Zhou Q. He Z. Q. Shen Z. F. Zhang W. X. Zhang D. (2020). Traditional Chinese medicine (liang-Xue-Di-huang decoction) for hemorrhoid hemorrhage: study protocol clinical trial (SPIRIT compliant). Medicine 99 (16), e19720. 10.1097/MD.0000000000019720 32311960 PMC7220198

[B78] Simbo D. J. (2010). An ethnobotanical survey of medicinal plants in babungo, northwest region, Cameroon. J. Ethnobiol. Ethnomed. 6 (1), 8. 10.1186/1746-4269-6-8 20156356 PMC2843657

[B79] Singh D. Siew Y. Yew H. Neo S. Koh H. (2019). “Botany, phytochemistry, and pharmacological activities of Leea species,” in Medicinal Plants: Chemistry, Pharmacology and Therapeutic Applications, 11–41. 10.1201/9780429259968-2

[B80] Sireeratawong S. Piyabhan P. Singhalak T. Wongkrajang Y. Temsiririrkkul R. Punsrirat J. (2011). Toxicity evaluation of sappan wood extract in rats. J. Med. Assoc. Thai. 93 (12), 50–S57. 21294398

[B81] Soladoye M. O. Adetayo M. O. Chukwuma E. C. Adetunji A. N. (2010). Ethnobotanical survey of plants used in the treatment of haemorrhoids in south-western Nigeria. Ann. Biol. Res. 1 (4), 1–15.

[B82] Sreekeesoon D. P. Mahomoodally M. F. (2014). Ethnopharmacological analysis of medicinal plants and animals used in the treatment and management of pain in Mauritius. J. Ethnopharmacol. 157, 181–200. 10.1016/j.jep.2014.09.030 25261690

[B83] Srinivasan G. Ranjith C. Vijayan K. (2008). Identification of chemical compounds from the leaves of *Leea indica* . Acta Pharm. 58 (2), 207–214. 10.2478/v10007-008-0002-7 18515230

[B84] Suwan T. Wanachantararak P. Khongkhunthian S. Okonogi S. (2018). Antioxidant activity and potential of *Caesalpinia sappan* aqueous extract on synthesis of silver nanoparticles. Drug Discov. Ther. 12 (5), 259–266. 10.5582/ddt.2018.01059 30464156

[B85] Tardío J. Pardo-de-Santayana M. Cortes Sánchez-Mata de (2016). “Ethnobotanical analysis of wild fruits and vegetables traditionally consumed in Spain,” in Mediterranean wild edible plants. Mediterranean wild edible plants. Editor Tardío J. (Springer), 57–79.

[B86] Tewtrakul S. Tungcharoen P. Sudsai T. Karalai C. Ponglimanont C. Yodsaoue O. (2015). Antiinflammatory and wound healing effects of Caesalpinia sappan L. Phytother. Res. 29 (6), 850–856. 10.1002/ptr.5321 25760294

[B87] Thangal A. H. Prasanth C. Prasanth M. Anu V. (2022). Pharmacognostic, phytochemical and pharmacological aspects of *Caesalpinia sappan* plant. Int. J. Pharm. Sci. Res. 10. 10.13040/IJPSR.0975-8232.IJP.9(12).213-19

[B88] Varma S. B. Giri S. P. (2013). Study of wound healing activity of Tectona grandis linn. Leaf extract on rats. Anc. Sci. Life. 32 (4), 241–244. 10.4103/0257-7941.131984 24991074 PMC4078476

[B89] Vidya S. Krishna V. Manjunatha B. Mankani K. Ahmed M. Singh S. (2007). Evaluation of hepatoprotective activity of *Clerodendrum serratum* L. Indian J. Exp. Biol. 45 (06), 538–542. 17585689

[B90] Vij T. Anil P. P. Shams R. Dash K. K. Kalsi R. Pandey V. K. (2023). A comprehensive review on bioactive compounds found in *Caesalpinia sappan* . Molecules 28 (17), 6247. 10.3390/molecules28176247 37687076 PMC10488625

[B91] Vyas P. Yadav D. K. Khandelwal P. (2019). *Tectona grandis* (Teak)–A review on its phytochemical and therapeutic potential. Nat. Prod. Res. 33 (16), 2338–2354. 10.1080/14786419.2018.1440217 29506390

[B92] Wong Y. H. Abdul Kadir H. Ling S. K. (2012). Bioassay-guided isolation of cytotoxic cycloartane triterpenoid glycosides from the traditionally used medicinal plant *Leea indica* . Evid. Based Complement. Altern. Med. 2012, 164689. 10.1155/2012/164689 22203865 PMC3235747

[B93] Xu H. Y. Zhang Y. Q. Liu Z. M. Chen T. Lv C. Y. Tang S. H. (2019). ETCM: an encyclopaedia of traditional Chinese medicine. Nucleic Acids Res. 47 (D1), D976–D982. 10.1093/nar/gky987 30365030 PMC6323948

[B94] Yan Y. Chen Y. C. Lin Y. H. Guo J. Niu Z. R. Li L. (2015). Brazilin isolated from the heartwood of *Caesalpinia sappan* L induces endothelium-dependent and-independent relaxation of rat aortic rings. Acta Pharmacol. Sin. 36 (11), 1318–1326. 10.1038/aps.2015.113 26564314 PMC4644902

[B95] Yao R. Heinrich M. Wei J. Xiao P. (2021). Cross-cultural ethnobotanical assembly as a new tool for understanding medicinal and culinary values–the genus lycium as A case study. Front. Pharmacol. 12, 708518. 10.3389/fphar.2021.708518 34335270 PMC8322658

[B96] Yao R. He C. Xiao P. (2023). Food and medicine continuum’in the East and West: old tradition and current regulation. Chin. Herb. Med. 15 (1), 6–14. 10.1016/j.chmed.2022.12.002 36875443 PMC9975626

[B97] Yao R. Gao J. Heinrich M. Yu S. Xue T. Zhang B. (2024). Medicinal plants used by minority ethnic groups in China: taxonomic diversity and conservation needs. J. Ethnopharmacol. 334, 118573. 10.1016/j.jep.2024.118573 38996945

[B98] Yemele M. Telefo P. Lienou L. Tagne S. Fodouop C. Goka C. (2015). Ethnobotanical survey of medicinal plants used for pregnant women׳ s health conditions in Menoua division-West Cameroon. J. Ethnopharmacol. 160, 14–31. 10.1016/j.jep.2014.11.017 25449451

[B99] Zhang J. Yuan K. Zhou W.-l. Zhou J. Yang P. (2011). Studies on the active components and antioxidant activities of the extracts of *Mimosa pudica* Linn. from southern China. Pharmacogn. Mag. 7 (25), 35–39. 10.4103/0973-1296.75899 21472077 PMC3065154

