# Supplementary material for: An ethnobotanical review of medicinal plants used for treating hemorrhoids in Thailand
Source: Front Pharmacol. 2026 Apr 24;17:1705134. doi: 10.3389/fphar.2026.1705134 (PMC13152847; doi:10.3389/fphar.2026.1705134)
Supplement: Supplementary file 1 [file Table1.docx]

**Supplementary Table 1** Characteristics of the 53 references of medicinal plans used to treat hemorrhoids in Thailand.

| **Study** | **Title** | **Province** | **Ethnic groups** | **No. of species** | **publication type** |
| --- | --- | --- | --- | --- | --- |
| Bunsongthae and Chaiwong, 2010 | Conservation and utilization of ethnic plants in some areas of Mae Hong Son province | Mae Hong Son | Karen, Thai hill tribe | 3 | Journal article |
| Chamratpan and Homchuen, 2003 | Ethnobotany in upper northeastern Thailand | Udon Thani, Ngong Kai, Ngong Bua Lampoo | Thai | 1 | Journal article |
| Chaunchom, 2011 | Ethnobotany of Hmong at Ban Tabboek, Tambon Wangban, Amphoe Lomkao, Phetchabun province | Phetchabun | Hmong | 1 | Thesis |
| Chotchoungchatchai et al., 2012 | Medicinal plants used with Thai traditional medicine in modern healthcare services: a case study in Kabchoeng Hospital, Surin province | Surin | Thai | 3 | Journal article |
| Chuakul, 2005 | Medicinal Plants in the Khok Pho District, Pattani province (Thailand) | Pattani | Thai | 5 | Journal article |
| Chuakul et al., 2004 | Survey on medicinal plants in southern Thailand | Surat Thani, Yala, Krabi, Satun, Chumphon | Thai | 1 | Journal article |
| Chuakul et al., 2006 | Medicinal plants used in Kungkrabaen Royal Development Study Center, Chanthaburi province | Chanthaburi | Thai | 3 | Journal article |
| Hanchanlert et al., 2005 | Exploration and collection of medicinal plants in Nakhon Ratchasima province | Nakhon Ratchasima | Thai | 1 | Proceeding |
| Hutasingha, 2015 | Ethnobotany of Akha in Mae La-ngong Village, Phrao District, Chiang Mai province | Chiang Mai | Akha | 1 | Thesis |
| Inta, 2008 | Ethnobotany and Crop Diversity of Tai Lue and Akha Communities in the Upper Northern Thailand and the Xishuangbanna Dai Autonomous Prefecture, China | Chiang Rai, Nan | Tai Lue, Akha | 7 | Thesis |
| Inta, 2014 | A Comparative Study of Ethnobotany of Ethnic groups in Pang Mapha District, Mae Hong Son province, Thailand | Mae Hong Son | Karen, Lahu, Lisu, Tai Yai | 2 | Book |
| Inta et al., 2011 | An analysis of knowledge on the utilization of local plants of Chiang Mai University Hariphunchai Education Centre, Lamphun province | Lamphun | Tai Yuan | 7 | Book |
| Inta et al., 2012 | Medicinal plants in Ban Hua Thung Community Forest, Chiang Dao Wildlife Sanctuary, Chang Dao district, Chiang Mai province | Chiang Mai | Tai Yuan | 30 | Journal article |
| Inta et al., 2013 | Analysis of traditional knowledge in medicinal plants used by Yuan in Thailand | Lamphun | Tai Yuan | 23 | Journal article |
| Jeanwitchayakul, 2014 | Diversity of Medicinal Plants and Local Wisdom in Kao Subkangkai Lopburi province | Lopburi | Thai | 2 | Proceeding |
| Junsongduang et al., 2018 | Dye plants and traditional knowledge of natural dyeing of Tai-Lao ethnicity in At Samat and Pho Chai districts, Roi Et province | Roi Et | Tai-Lao | 1 | Journal article |
| Kadchumsang et al., 2015 | Antibacterial and antioxidant activities of Lanna medicinal plants used in Mahoog formula | Chiang Mai, Chiang Rai, Lampang | Tai Yuan | 23 | Journal article |
| Kamwong, 2009 | Ethnobotany of Karens at Ban Mai Sawan and Ban Huay Pu Ling, Ban Luang Sub-District, Chom Thong District, Chiang Mai province | Chiang Mai | Karen | 5 | Thesis |
| Leeratiwong et al., 2016 | Ethnobotanical study in Ko Hong Hill, Songkhla province | Songkhla | Thai | 6 | Journal article |
| Maneenoon et al., 2015 | Ethnomedicinal plants used by traditional healers in Phatthalung province, Peninsular Thailand | Phatthalung | Thai | 3 | Journal article |
| Muangyen, 2013 | Ethnobotany of Tai Lue and Tai Yuan in Samoeng District, Chiang Mai province | Chiang Mai | Tai Lue, Tai Yuan | 4 | Thesis |
| Neamsuvan et al., 2015 | A survey of medicinal plants around upper Songkhla Lake, Thailand | Songkhla | Thai | 4 | Journal article |
| Nguanchoo, 2014 | Ethnobotany of Hmong in Mae Rim district, Chiang Mai province, Thailand | Chiang Mai | Hmong | 10 | Thesis |
| Osiri et al., 2001 | Folk healers and herbal use in Chonburi province | Chonburi | Thai | 1 | Journal article |
| Panyadee, 2012 | Plant diversity in homegardens of Tai Yai communities in Pang Mapha district, Mae Hong Son province | Mae Hong Son | Tai Yai | 1 | Thesis |
| Panyadee, 2017 | Comparison of Plants Composition and Structure of Homegardens of Ethnic Groups in Chiang Mai province, Thailand | Chiang Mai | Thai Yuan, Hmong | 2 | Thesis |
| Phatlamphu et al., 2021 | Ethnobotany of edible plants in Muang district, Kalasin province, Thailand | Kalasin | Thai Lao | 4 | Journal article |
| Phongloy, 2015 | Biodiversity and Utilization of Plants from Protected and Utilized Forests by Tai Yai Communities in Chiang Mai and Mae Hong Son province | Chiang Mai, Mae Hong Son | Tai Yai | 4 | Thesis |
| Pipitkul, 2001 | Medicinal Plant Utilization for the Living of Hill Tribes at Doi Musoe, Tak province | Tak | Lahu | 1 | Thesis |
| Pongamornkul, 2006 | An Ethnobotanical Study of Lua in Royal Project Areas, Mae Hong Son province | Mae Hong Son | Lua | 2 | Thesis |
| Pongamornkul, 2010 | Annual report of the project collecting and survey local plants and Lanna herbs | - | Lanna | 2 | Report |
| Pongamornkul and Muangyen, 2012 | Ethnobotany of Tai Yai in Khun Yuam District, Mae Hong Son province | Mae Hong Son | Tai Yai | 12 | Report |
| Pongamornkul and Muangyen, 2013 | Ethnobotany of Karen in Sop Moei District, Mae Hong Son province | Mae Hong Son | Karen | 1 | Report |
| Pongamornkul, 2009 | Annual report of the project collecting and exhibiting local plants and Lanna herbs | - | Tai Yuan | 18 | Report |
| Pongsattayapipat, 1999 | Survey and Collection of Cereals, Legumes and Earth Crops Consumed by the Minorities in Some Areas of Doi Mae Salong in Chiang Rai Province | Chiang Mai | Karen | 1 | Thesis |
| Ponpim, 1996 | Ethnobotany of the hill tribes in Kaenoy's and Nongkheuw's Royal Project in Chiang Mai | Chiang Mai | Lahu, Lawa | 2 | Thesis |
| Purintavaragul et al., 2012 | Medicinal plants diversity in Kao-Pra village, Kao-Pra subdistrict, Rattapoom district, Songkhla province | Songkhla | Thai | 5 | Journal article |
| Sinworn and Viriyawattana, 2014 | The Diversity of Medicinal Plants and Utilization in Khoa Phra, Doembangnangbuat district, Suphanburi province | Suphanburi | Thai | 3 | Journal article |
| Songsangchun, 2015 | Plants Usages of Khon Muang and Lawa in Phu Fah subdistrict, Bo Klua district, Nan province | Nan | Tai Yuan, Lawa | 1 | Thesis |
| Sonsupub, 2010 | Ethnobotany of Karen Community in Raipa Village, Huaykhayeng subdistrict, Thongphaphume district, Kanchanaburi province | Kanchanaburi | Karen | 1 | Thesis |
| Srisanga, 1993 | The ethnobotany of Hmong Lai in Mae Sa Mai village in Chiang Mai province | Chiang Mai | Hmong Lai | 3 | Thesis |
| Srithi, 2012 | Comparative Ethnobotany in Nan province, Thailand | Nan | Mien, Khamu | 8 | Thesis |
| Sukkho, 2008 | A survey of medicinal plants used by Karen People at Ban Chan and Chaem Luang subdistricts, Mae Chaem district, Chiang Mai province | Chiang Mai | Karen | 8 | Thesis |
| Sumridpiem, 2017 | Utilization Analysis of Medicinal Plants Among Tai Yong and Tai Yuan in Lamphun province | Lamphun | Tai Yong, Tai Yuan | 33 | Thesis |
| Tangjitman, 2017 | Ethnobotany of the Karen at Huay Nam Nak village, Tanaostri subdistrict, Suanpheng district, Ratchaburi province | Ratchaburi | Karen | 1 | Journal article |
| Tangtragoon, 1998 | Ethnobotany of the Khamu, Lawa and H'tin in Some Areas of Nan province | Nan | Khamu, Lawa, H'tin | 1 | Thesis |
| Tangtragoon et al., 2004 | Ethnobotany Studies in Ban Pong, Sansai District, Chiang Mai province | Chiang Mai | Tai Yai | 1 | Book |
| Tovaranonte, 2001 | Ethnobotany in Surroundings Area of Mae Fah Luang University | Chiang Rai | Lanna | 5 | Report |
| Tovaranonte, 2003 | Ethnobotany of Tai Lue in Chiang Rai province | Chiang Rai | Tai Lue | 1 | Report |
| Trisonthi and Trisonthi, 2011 | Ethnobotany of Lua and H’tin on Doi Phukha, Nan province | Nan | Lua and H’ tin | 2 | Journal article |
| Trisonthi et al., 2007 | Research Project on Gathering of Highland Traditional Biodiversity- and Ethnobiology-Based Knowledge | - | Thai Yuan, Shan | 7 | Report |
| Winjchiyanan, 1995 | Ethnobotany of Karen in Chiang Mai | Chiang Mai | Karen | 8 | Thesis |
| Yaso, 1997 | Ethnobotany of Black Lahu in Huai Pong Village, Wiang Pa Pao District, Chaing Mai province | Chiang Mai | Black Lahu | 3 | Thesis |

**References**

Bunsongthae, A., and Chaiwong, C. (2010). Conservation and utilization of ethnic plants in some areas of Maehongson province. *North. Reg. J. Sci. Technol* 3**,** 22–43.

Chamratpan, S., and Homchuen, S.-a. (2003). Ethnobotany in upper northeastern Thailand. *Acta Hortic.* 675**,** 67–74.

Chaunchom, P. (2011). *Ethnobotany of Hmong at Ban Tabboek, Tambon Wangban, Amphoe Lomkao, Changwat Phetchabun.* Master of Science, Kasetsart University, Bangkok, Thailand.

Chotchoungchatchai, S., Saralamp, P., Jenjittikul, T., Pornsiripongse, S., and Prathanturarug, S. (2012). Medicinal plants used with Thai Traditional Medicine in modern healthcare services: A case study in Kabchoeng Hospital, Surin Province, Thailand. *J. Ethnopharmacol.* 141(1)**,** 193–205.

Chuakul, W. (2005). Medicinal plants in the Khok Pho district, pattani province (Thailand).

Chuakul, W., Soonthornchareonnon, N., Boonjaras, T., and Boonpleng, A. (2004). Survey on medicinal plants in Southern Thailand. *Thai J. Phytopharm.* 11**,** 2.

Chuakul, W., Soonthornchareonnon, N., and Sappakun, S. (2006). Medicinal plants used in Kungkrabaen royal development study center, Chanthaburi province. *Thai J. Phytopharm.* 13(1)**,** 27–42.

Hanchanlert, O. A., Babpraserth, C., and Paisooksantivatana, Y. (2005). "Exploration and collection of medicinal plants in Nakhon Ratchasima province", in: *Thai Resources Conference: All things are Connected.*, 504–510.

Hutasingha, P. (2015). *Ethnobotany of Akha in Mae La-ngong Village, Namphrae Subdistrict, Phrao District, Chiang Mai Province.* Bachelor's degree, Chiang Mai University.

Inta, A. (2008). *Ethnobotany and crop diversity of Tai Lue and Akha communities in the upper northern Thailand and the Xishuangbanna Dai autonomous prefecture, China.* Chiang Mai: Graduate School, Chiang Mai University, 2008.

Inta, A. (2014). *A Comparative Study of Ethnobotany of Ethnic groups in Pang Mapha District, Maehongson Province.* Chiang Mai, Thailand: Department of Biology, Faculty of Science, Chiang Mai University.

Inta, A., Sirisa-ard, P., and Pongamornkul, W. (2012). Medicinal plants in Ban Hua Thung Community Forest, Chiang Dao Wildlife Sanctuary, Chang Dao district, Chiang Mai province. *Thai J. Bot.* 4**,** 213–232.

Inta, A., Trisonthi, C., and Trisonthi, P. (2011). *An analysis of knowledge on the utilization of local plants of Chiang Mai University Hariphunchai Education Centre,Lamphun Province, Chiang Mai University.* Chiang Mai, Thailand: Department of Biology, Faculty of Science, Chiang Mai University.

Inta, A., Trisonthi, P., and Trisonthi, C. (2013). Analysis of traditional knowledge in medicinal plants used by Yuan in Thailand. *J. Ethnopharmacol.* 149(1)**,** 344–351.

Jeanwitchayakul, P. (2014). "Diversity of Medicinal Plants and Local Wisdom in Kao Subkangkai Lopburi Province", in: *The 6th natioal MCRU meeting on Thailand sustainable development*.

Junsongduang, A., Sirithip, K., Nachai, R., Buakamkoat, L., and Ornputtha, B. (2018). Dye plants and traditional knowledge of natural dyeing of Tai-Lao ethnicity in At Samat and Pho Chai districts, Roi Et province. *Thai J. Bot.* 2018*.*

Kadchumsang, S., Sirisa-Ard, P., Sookkhee, S., and Chansakaow, S. (2015). Antibacterial and antioxidant activities of lanna medicinal plants used in mahoog formula. *Int. J. Pharm. Pharm. Sci.* 7(9)**,** 366–370.

Kamwong, K. (2009). *Ethnobotany of Karens at Ban Mai Sawan and Ban Huay Pu Ling, Ban Luang Sub-District, Chom Thong District, Chiang Mai Province.* Master of Science (Biology), Chiang Mai University.

Leeratiwong, C., Maneenoon, K., and Sawangchote, P. (2016). Ethnobotanical study in Ko Hong hill, Songkhla province. *Thai J. Bot.* 2016*.*

Maneenoon, K., Khuniad, C., Teanuan, Y., Saedan, N., Prom-In, S., Rukleng, N., et al. (2015). Ethnomedicinal plants used by traditional healers in Phatthalung Province, Peninsular Thailand. *J. Ethnobiol. Ethnomed.* 11(1)**,** 1–20.

Manyaem, S., Namsa-ard, M., Puttikamonkul, S., Prachya, S., Namsa-ard, A., and Samosorn, S. (2022). Chemical Constituents and their Antifungal Activity from the Branch Extracts of *Plumbago indica* L. *J Chulabhorn Royal Acad.* 4(4)**,** 189–197.

Nguanchoo, V. (2014). Ethnobotany of Hmong in Mae Rim District, Chiang Mai Province, Thailand. *Master of Science Thesis in Plant Science, Graduate Studies, Mahidol University*.

Osiri, S., Matchacheep, S., Thalerngpong, J., Mudlee, N., Noiprasert, N., and Patanapokratana, P. (2001). Folk healers and herbal use in Chonburi Province. *Public Health J. Burapha U.* 6**,** 53–62.

Panyadee, P. (2012). *Plant Diversity in Homegardens of Tai Yai Communities in Pang Mapha District, Mae Hong Son Province.* Master of science, Chiang Mai University.

Panyadee, P. (2017). *Comparison of Plants Composition and Structure of Homegardens of Ethnic Groups in Chiang Mai Province, Thailand.* Doctor of Philosophy (Biodiversity and Ethnobiology), Chiang Mai University.

Phatlamphu, N., Saensouk, S., Saensouk, P., and Junsongduang, A. (2021). Ethnobotany of edible plants in Muang District, Kalasin Province, Thailand. *Biodiversitas.* 22(12)**,** 5432–5444.

Phongloy, T. (2015). *Biodiversity and Utilization of Plants from Protected and Utilized Forests by Tai Yai Communities in Chiang Mai and Mae Hong Son Province.* Doctor of Philosophy (Biodiversity and Ethnobioloy), Chiang Mai University.

Pipitkul, S. (2001). *Medicinal Plant Utilization for the Living of Hill Tribes at Doi Musoe, Tak Province.* Master of Science, Mahidol University.

Pongamornkul, W. (2006). *An Ethnobotanical study of Lua in royal project areas, Mae Hong Son Province.* Master of Science (Biology), Chiang Mai University.

Pongamornkul, W. (2009). "Annual report of the project collecting and exhibiting local plants and Lanna herbs; Annual report in 2009", (ed.) Q.S.B. Garden. (Chiang Mai, Thailand: Queen Sirikit Botanical Garden ).

Pongamornkul, W. (2010). "Annual report of the project collecting and servey local plants and Lanna herbs; Annual report in 2010"", (ed.) Q.S.B. Garden. (Chiang Mai, Thailand: Queen Sirikit Botanical Garden ).

Pongamornkul, W. (2017). *Northern Thailand Ethnobotanical index.* Thailand: Wanida Karnpim Limited Partmership.

Pongamornkul, W., and Muangyen, N. (2012). *Ethnobotany of Tai Yai in Khun Yuam District, Mae Hong Son province. Annual report in 2013.The ethbobotany of ethnic groups in Nortern of Thailand.* Chiang Mai, Thailand: The Botanical Garden Organization, Ministry of Natural Resources and Environment.

Pongamornkul, W., and Muangyen, N. (2013). *Ethnobotany of Karen in Sop moei district, Mae Hong Son province. Annual report in 2013.The ethbobotany of ethnic groups in Nortern of Thailand. .* Chiang Mai, Thailand: Queen Sirikit Botanical Garden.

Pongsattayapipat, R. (1999). *Survey and Collection of Cereals, Legumes and Earth Crops Consumed by the Minorities in Some Areas of Doi Maw Salong in Chiang Rai Province.* Master degree, Chiang Mai University.

Ponpim, Y. (1996). *Ethnobotany of the Hill Tribes in Kaenoy's and Nongkheuw's Royal Project in Chiang Mai.* Master of science, Chiang Mai University.

Purintavaragul, C., Wongnawa, M., and Thaina, P. (2012). Medicinal plants diversity in Kao-Pra village, Thumbol Kao-Pra, Amphoe Rattapoom, Songkhla Province. *Thaksin J.* 15(9).

Sinworn, S., and Viriyawattana, N. (2014). The diversity of medicinal plants and utilization in Khoa Phra, Doembangnangbuat District, Suphanburi Province. *SDU Res. J. Sci. Tech.* 7**,** 19.

Songsangchun, A. (2015). *Plants Usages of Khon Muang and Lawa in Phu Fah Subdistrict, Bo Klua District, Nan Province.* Master of science, Chiang Mai University.

Sonsupub, B. (2010). *Ethnobotany of Karen Community in Raipa Village, Huaykhayeng Subdistrict, Thongphaphume District, Kanchanaburi Province.* Master of Science (Agriculture), Kasetsart University, Bankok, Thailand.

Srisanga, P. (1993). *The ethnobotany of Hmong Lai in Mae Sa Mai village in Chiang Mai Province.* Bachelor's degree, Chiang Mai University.

Srithi, K. (2012). *Comparative ethnobotany in Nan province, Thailand.* Doctor of Philosophy, Chiang Mai University.

Sukkho, T. (2008). *A Survey of Medicinal Plants Used by Karen People at Ban Chan and Chaem Luang Subdistricts, Mae Chaem District, Chiang Mai Province.* Master of Science (Biology), Chiang Mai University, Chiang Mai.

Sumridpiem, P. (2017). *Utilization Analysis of Medicinal Plants Among Tai Yong And Tai Yuan in Lamphun Province.* Master of Science (Biology), Chiang Mai University.

Tangjitman, K. (2017). Ethnobotany of the Karen at Huay Nam Nak village, Tanaosri subdistrict, Suanphueng district, Ratchaburi province. *Thai J. Bot.* 9(2)**,** 253–272.

Tangtragoon, T. (1998). *Ethnobotany of the Khamu, Lawa and H'tin in Some Areas of Nan Province.* Master of Science, Chiang mai University.

Tangtragoon, T., Tarachai, Y., Hongwittayakorn, P., Prokati, V., and Phreechawattanakon, P. (2004). "Ethnobotany Studies in Ban Pong, Sansai District, Chiang Mai Province. Retrieved from https://www.rspg.or.th/articles/e_book/

Tovaranonte, J. (2001). "Ethnobotany in Surroundings Area of Mae Fah Luang University. School of Science, Mae Fah Luang University". (Chiang Rai: Mae Fah Luang University).

Tovaranonte, J. (2003). "Ethnobotany of Tai Lue in Chiang Rai Province". (Chiang Rai: School of Science, Mae Fah Luang University.).

Trisonthi, C., and Trisonthi, P. (2011). Ethnobotany of Lua and H'tin on Doi Phukha, Nan province. *Thai J. Bot.* 3(2), 163–185.

Trisonthi, C., Trisonthi, P., Wangpakapattanawong, P., and Srisanga, P. (2007). "Research Project on Gathering of Highland Traditional Biodiversity- and Ethnobiology-Based Knowledge". (Chiang Mai: Highland Research and Development Institute (Public Organization)).

Winjchiyanan, P. (1995). *Ethnobotany of Karen in Chiang Mai.* Master of Science (Biology), Chiang Mai University.

Yaso, T. (1997). *Ethnobotany of Black Lahu in Huai Pong Village, Wiang Pa Pao District, Chaiang Mai Province.* Bachelor of Science, Chiang Mai University.
