# Supplementary material for: An ethnobotanical review of medicinal plants used for treating hemorrhoids in Thailand
Source: Front Pharmacol. 2026 Apr 24;17:1705134. doi: 10.3389/fphar.2026.1705134 (PMC13152847; doi:10.3389/fphar.2026.1705134)
Supplement: Supplementary file 2 [file Table2.docx]

**Supplementary Table 2 Medicinal plants used to treat hemorrhoids in Thailand.**

| **Family** | **Scientific name** | **Plant part used** | **Preparation** | **Application** | **UV** | **CV** | **Reference** |
| --- | --- | --- | --- | --- | --- | --- | --- |
| Acanthaceae | *Phlogacanthus curviflorus* (Neeraja et al.) Nees | root | decoction | oral ingestion | 0.02 | 0.01 | Kamwong, 2009 |
|  | *Rhinacanthus nasutus* (L.) Kurz | whole plants | concoction | oral ingestion | 0.02 | 0.01 | Sumridpiem, 2017 |
|  | *Thunbergia grandiflora* Roxb. | root | decoction | oral ingestion | 0.02 | 0.01 | Tovaranonte, 2001 |
|  | *Thunbergia laurifolia* Lindl. | leaf, stem, whole plants | concoction, decoction | bath, oral ingestion, poultice, sauna | 0.06 | 0.03 | Inta et al., 2011; Inta et al., 2013; Kadchumsang et al., 2015 |
| Amaranthaceae | *Amaranthus spinosus* L. | root, whole plants | decoction | oral ingestion | 0.06 | 0.03 | Tovaranonte, 2001; Pongamornkul and Muangyen, 2012; Songsangchun, 2015 |
|  | *Spondias pinnata* (L.f.) Kurz | fruit, root, stem | decoction, none | bath, oral ingestion, poultice, sauna | 0.04 | 0.02 | Inta et al., 2011; Inta et al., 2013 |
| Ancistrocladaceae | *Ancistrocladus tectorius* (Lour.) Merr. | stem, wood | decoction | oral ingestion | 0.02 | 0.01 | Leeratiwong et al., 2016 |
| Annonaceae | *Uvaria dulcis* Dunal | stem | concoction | oral ingestion | 0.02 | 0.01 | Kadchumsang et al., 2015 |
|  | *Uvaria ferruginea* var. *cherrevensis* (Pierre ex Finet & Gagnep.) Meade & J.Parn. | root | decoction | bath, oral ingestion, poultice, sauna | 0.04 | 0.02 | Inta et al., 2011; Inta et al., 2013 |
| Apiaceae | *Anethum graveolens* L. | seed | concoction, cooked, eaten as food, pill | oral ingestion | 0.06 | 0.01 | Sumridpiem, 2017 |
|  | *Angelica dahurica* (Hoffm.) Benth. & Hook.f. ex Franch. & Sav. | root | cooked, none, pill | oral ingestion | 0.04 | 0.01 | Sumridpiem, 2017 |
|  | *Foeniculum vulgare* Mill. | seed | cooked, none | oral ingestion | 0.02 | 0.01 | Sumridpiem, 2017 |
| Apocynaceae | *Alstonia scholaris* (L.) R.Br. | bark | decoction | oral ingestion | 0.04 | 0.02 | Winjchiyanan, 1995; Pongamornkul and Muangyen, 2012 |
|  | *Amphineurion marginatum* (Roxb.) D.J.Middleton | stem | concoction | oral ingestion | 0.02 | 0.01 | Kadchumsang et al., 2015 |
|  | *Cryptolepis buchananii* R.Br. ex Roem. & Schult. | stem | not specified | not specified | 0.02 | 0.01 | Pongamornkul, 2009 |
|  | *Plumeria obtusa* L. | stem | not specified | not specified | 0.02 | 0.01 | Pongamornkul, 2009 |
|  | *Streptocaulon juventas* (Lour.) Merr. | root | decoction | bath | 0.02 | 0.01 | Inta et al., 2011 |
| Araceae | *Alocasia longiloba* Miq. | rhizome | decoction | oral ingestion | 0.02 | 0.01 | Leeratiwong et al., 2016 |
|  | *Alocasia macrorrhizos* (L.) G.Don | rhizome | decoction | oral ingestion | 0.02 | 0.01 | Leeratiwong et al., 2016 |
|  | *Colocasia esculenta* (L.) Schott | rhizome | not specified | not specified | 0.02 | 0.01 | Pongamornkul and Muangyen, 2012 |
|  | *Pothos chinensis* (Raf.) Merr. | leaf, whole plants | concoction | oral ingestion | 0.02 | 0.01 | Trisonthi et al., 2007 |
| Arecaceae | *Areca catechu* L. | seed | pill | oral ingestion | 0.02 | 0.01 | Sumridpiem, 2017 |
|  | *Borassus flabellifer* L. | root | concoction, decoction | oral ingestion | 0.04 | 0.02 | Inta et al., 2011; Inta et al., 2013 |
|  | *Cocos nucifera* L. | fruit, root | decoction, none | oral ingestion | 0.04 | 0.02 | Inta et al., 2011; Inta et al., 2013 |
| Asparagaceae | *Asparagus racemosus* Willd. | root | rub with lime water | topical treatment | 0.02 | 0.01 | Chuakul et al., 2006 |
|  | *Cordyline fruticosa* (L.) A.Chev. | flower, stem | concoction | oral ingestion | 0.02 | 0.01 | Winjchiyanan, 1995 |
| Asphodelaceae | *Aloe vera* (L.) Burm.f. | leaf | none | topical treatment | 0.02 | 0.01 | Sumridpiem, 2017 |
|  | *Dianella ensifolia* (L.) Redouté | leaf | pill | oral ingestion | 0.02 | 0.01 | Inta, 2008 |
| Asteraceae | *Acmella oleracea* (L.) R.K.Jansen | whole plants | decoction | oral ingestion | 0.04 | 0.01 | Pongamornkul, 2006 |
|  | *Blumea balsamifera* (L.) DC. | leaf | decoction | oral ingestion | 0.04 | 0.02 | Ponpim, 1996; Tovaranonte, 2001 |
|  | *Chromolaena odorata* (L.) R.M.King & H.Rob. | root | decoction | oral ingestion | 0.02 | 0.01 | Inta, 2014 |
|  | *Duhaldea cappa* (Buch.-Ham. ex D.Don) Pruski & Anderb. | whole plants | decoction | oral ingestion | 0.02 | 0.01 | Tovaranonte, 2001 |
|  | *Eclipta prostrata* (L.) L. | leaf | decoction, pounding | oral ingestion, poultice | 0.04 | 0.02 | Inta et al., 2011; Inta et al., 2013 |
|  | *Elephantopus scaber* L. | whole plants | concoction | oral ingestion | 0.02 | 0.01 | Winjchiyanan, 1995 |
|  | *Pluchea indica* (L.) Less. | bark, leaf, whole plants | decoction, squash with water | oral ingestion | 0.06 | 0.03 | Osiri et al., 2001; Neamsuvan et al., 2015; Phatlamphu et al., 2021 |
| Betulaceae | *Betula alnoides* Buch.-Ham. ex D.Don | stem | decoction | oral ingestion | 0.02 | 0.01 | Trisonthi et al., 2007 |
| Bignoniaceae | *Millingtonia hortensis* L.f. | flower, root, stem, wood | concoction | oral ingestion | 0.06 | 0.03 | Tangtragoon et al., 2004; Kadchumsang et al., 2015; Sumridpiem, 2017 |
| Bixaceae | *Bixa orellana* L. | root, stem | concoction | oral ingestion | 0.02 | 0.01 | Sumridpiem, 2017 |
| Boraginaceae | *Heliotropium indicum* L. | whole plants | concoction | oral ingestion | 0.02 | 0.01 | Pongamornkul and Muangyen, 2012 |
| Cannaceae | *Canna indica* L. | rhizome | decoction | oral ingestion | 0.02 | 0.01 | Nguanchoo, 2014 |
| Capparaceae | *Crateva magna* (Lour.) DC. | bark | decoction | oral ingestion | 0.02 | 0.01 | Neamsuvan et al., 2015 |
| Caricaceae | *Carica papaya* L. | stem | concoction | oral ingestion | 0.02 | 0.01 | Sumridpiem, 2017 |
| Celastraceae | *Celastrus paniculatus* Willd. | stem | concoction | oral ingestion | 0.02 | 0.01 | Kadchumsang et al., 2015 |
|  | *Salacia chinensis* L. | stem | not specified | not specified | 0.02 | 0.01 | Phatlamphu et al., 2021 |
|  | *Siphonodon celastrineus* Griff. | stem | not specified | not specified | 0.02 | 0.01 | Pongamornkul, 2009 |
| Combretaceae | *Combretum deciduum* Collett & Hemsl. | stem | concoction | oral ingestion | 0.02 | 0.01 | Kadchumsang et al., 2015 |
|  | *Terminalia bellirica* (Gaertn.) Roxb. | stem | concoction | oral ingestion | 0.02 | 0.01 | Kadchumsang et al., 2015 |
|  | *Terminalia chebula* Retz. | fruit, leaf galls | capsule, grind and pill | oral ingestion | 0.04 | 0.02 | Chotchoungchatchai et al., 2012; Sumridpiem, 2017 |
| Commelinaceae | *Murdannia loriformis* (Hassk.) R.S.Rao & Kammathy | leaf | cooked with egg, eaten as food | oral ingestion | 0.04 | 0.02 | Srithi, 2012; Panyadee, 2017 |
| Convolvulaceae | *Cuscuta reflexa* Roxb. | root, whole plants | decoction | oral ingestion | 0.04 | 0.02 | Inta et al., 2011; Inta et al., 2013 |
| Cornaceae | *Alangium hexapetalum* Lam. | wood | not specified | not specified | 0.02 | 0.01 | Hanchanlert et al., 2005 |
| Cucurbitaceae | *Momordica cochinchinensis* (Lour.) Spreng. | root | not specified | not specified | 0.02 | 0.01 | Sinworn and Viriyawattana, 2014 |
|  | *Trichosanthes scabra* Lour. | seed | concoction | bath | 0.02 | 0.01 | Sukkho, 2008 |
| Dioscoreaceae | *Dioscorea bulbifera* L. | tuber | cooked, decoction | oral ingestion | 0.02 | 0.01 | Inta et al., 2012 |
|  | *Tacca plantaginea* (Hance) Drenth | root | pill | oral ingestion | 0.02 | 0.01 | Inta, 2008 |
| Dipterocarpaceae | *Dipterocarpus obtusifolius* Teijsm. ex Miq. | exudates, root | concoction, water infusion | oral ingestion | 0.04 | 0.02 | Inta et al., 2011; Inta et al., 2013 |
|  | *Hopea odorata* Roxb. | wood | pounding | topical treatment | 0.02 | 0.01 | Chuakul, 2005 |
|  | *Shorea obtusa* Wall. ex Blume | stem | concoction | oral ingestion | 0.02 | 0.01 | Kadchumsang et al., 2015 |
| Ebenaceae | *Diospyros* sp. | bark | decoction | soak | 0.02 | 0.01 | Inta et al., 2012 |
|  | *Diospyros variegata* Kurz | root, stem | decoction | oral ingestion | 0.02 | 0.01 | Jeanwitchayakul, 2014 |
| Elaeagnaceae | *Elaeagnus latifolia* L. | root | not specified | not specified | 0.02 | 0.01 | Pongamornkul and Muangyen, 2012 |
| Equisetaceae | *Equisetum ramosissimum* Desf. subsp. *huegelii* (Iannarelli et al.) Christenh. & Husby | whole plants | decoction | oral ingestion | 0.02 | 0.01 | Srithi, 2012 |
| Ericaceae | *Vaccinium sprengelii* (G.Don) Sleumer ex Rehder | root | concoction | oral ingestion | 0.02 | 0.01 | Kamwong, 2009 |
| Erythroxylaceae | *Erythroxylum cuneatum* (Miq.) Kurz | root | concoction | oral ingestion | 0.02 | 0.01 | Kadchumsang et al., 2015 |
| Euphorbiaceae | *Baliospermum solanifolium* (Burm.) Suresh | leaf, root | burn, decoction | oral ingestion, topical treatment | 0.02 | 0.01 | Inta et al., 2012 |
|  | *Croton crassifolius* Geiseler | root, stem | concoction | not specified, oral ingestion | 0.04 | 0.02 | Pongamornkul, 2009; Kadchumsang et al., 2015 |
|  | *Croton persimilis* Müll.Arg. | bark, exudates, leaf, root, stem | concoction, decoction, none, rub with stone and soke in water | bath, oral ingestion, poultice, sauna | 0.09 | 0.04 | Winjchiyanan, 1995; Yaso, 1997; Inta et al., 2011; Inta et al., 2013 |
|  | *Croton tiglium* L. | root, seed oil | decoction | oral ingestion | 0.02 | 0.01 | Maneenoon et al., 2015 |
|  | *Euphorbia hirta* L. | whole plants | grind, squash and added sugar | oral ingestion | 0.04 | 0.02 | Sinworn and Viriyawattana, 2014; Sumridpiem, 2017 |
|  | *Euphorbia tirucalli* L. | stem | dried and decoction, grind, pulped | oral ingestion, topical treatment | 0.06 | 0.03 | Tovaranonte, 2001; Inta, 2008; Srithi, 2012 |
|  | *Ricinus communis* L. | leaf | burn, decoction | oral ingestion, topical treatment | 0.06 | 0.01 | Nguanchoo, 2014 |
|  | *Shirakiopsis indica* (Willd.) Esser | leaf | decoction | oral ingestion | 0.02 | 0.01 | Neamsuvan et al., 2015 |
|  | *Trigonostemon reidioides* (Kurz) Craib | root | concoction | oral ingestion | 0.02 | 0.01 | Kadchumsang et al., 2015 |
| Fabaceae | *Biancaea sappan* (L.) Tod. | heart wood, stem | concoction, decoction, grind | oral ingestion | 0.11 | 0.06 | Pongamornkul, 2009; Inta et al., 2011; Inta et al., 2013; Kadchumsang et al., 2015; Sumridpiem, 2017; Phatlamphu et al., 2021 |
|  | *Brachypterum scandens* (Roxb.) Wight & Arn. ex Miq. | stem | concoction | oral ingestion | 0.02 | 0.01 | Kadchumsang et al., 2015 |
|  | *Caesalpinia pulcherrima* (L.) Sw. | stem | concoction | oral ingestion | 0.02 | 0.01 | Muangyen, 2013 |
|  | *Cassia fistula* L. | bark, fruit, leaf, stem | concoction, decoction, pounding | bath, not specified, oral ingestion | 0.06 | 0.03 | Pongamornkul, 2009; Inta et al., 2011; Inta et al., 2013 |
|  | *Dendrolobium thorelii* (Gagnep.) Schindl. | root, stem | decoction | oral ingestion | 0.02 | 0.01 | Inta et al., 2012 |
|  | *Mimosa pudica* L. | leaf, root, whole plants | burn, concoction, decoction | oral ingestion, topical treatment | 0.09 | 0.04 | Sukkho, 2008; Inta et al., 2011; Pongamornkul and Muangyen, 2012; Tangjitman, 2017 |
|  | *Pterocarpus macrocarpus* Kurz | stem | concoction | oral ingestion | 0.02 | 0.01 | Kadchumsang et al., 2015 |
|  | *Pterolobium macropterum* Kurz | stem | decoction | oral ingestion | 0.02 | 0.01 | Inta et al., 2012 |
|  | *Senegalia comosa* (Gagnep.) Maslin, Seigler & Ebinger | root, stem | pounding | oral ingestion | 0.02 | 0.01 | Inta et al., 2012 |
|  | *Senegalia rugata* (Lam.) Britton & Rose | leaf | pill | oral ingestion | 0.02 | 0.01 | Inta, 2008 |
|  | *Senna alata* (L.) Roxb. | stem, whole plants | decoction, dried and decoction | oral ingestion | 0.06 | 0.03 | Winjchiyanan, 1995; Pongamornkul, 2009; Pongamornkul and Muangyen, 2012 |
|  | *Senna alexandrina* Mill. | leaf | capsule | oral ingestion | 0.02 | 0.01 | Chotchoungchatchai et al., 2012 |
|  | *Senna siamea* (Lam.) H.S.Irwin & Barneby | bark, leaf, root, stem | concoction, decoction | oral ingestion | 0.06 | 0.03 | Pongamornkul, 2009; Inta et al., 2011; Inta et al., 2013 |
|  | *Sindora siamensis* Teijsm. ex Miq. | stem | concoction | oral ingestion | 0.02 | 0.01 | Kadchumsang et al., 2015 |
|  | *Tadehagi triquetrum* (L.) H.Ohashi | leaf, root | concoction, decoction | oral ingestion | 0.06 | 0.03 | Pongamornkul, 2010; Trisonthi and Trisonthi, 2011; Srithi, 2012 |
| Hernandiaceae | *Illigera appendiculata* Blume | not specified | decoction, dried | oral ingestion | 0.02 | 0.01 | Purintavaragul et al., 2012 |
| Hypericaceae | *Cratoxylum formosum* subsp. *pruniflorum* (Kurz) Gogelein | bark, shoot | decoction | oral ingestion | 0.02 | 0.01 | Ponpim, 1996 |
| Iridaceae | *Eleutherine bulbosa* (Mill.) Urb. | bulb | not specified | not specified | 0.02 | 0.01 | Purintavaragul et al., 2012 |
| Lamiaceae | *Clerodendrum chinense* (Osbeck) Mabb. | leaf, root | burn, decoction | bath, oral ingestion, topical treatment | 0.08 | 0.01 | Nguanchoo, 2014 |
|  | *Clerodendrum colebrookianum* Walp. | leaf | burn | topical treatment | 0.02 | 0.01 | Chaunchom, 2011 |
|  | *Clerodendrum infortunatum* L. | leaf | fresh | topical treatment | 0.02 | 0.01 | Pongamornkul and Muangyen, 2013 |
|  | *Clerodendrum japonicum* (Thunb.) Sweet | leaf, root, stem | burn, concoction | oral ingestion, topical treatment | 0.06 | 0.03 | Trisonthi et al., 2007; Bunsongthae and Chaiwong, 2010; Muangyen, 2013 |
|  | *Clerodendrum paniculatum* L. | flower, leaf | decoction, fresh | oral ingestion | 0.02 | 0.01 | Phongloy, 2015 |
|  | *Clerodendrum petasites* (Lour.) S.Moore | stem | not specified | not specified | 0.02 | 0.01 | Panyadee, 2012 |
|  | *Clerodendrum villosum* Blume | leaf | decoction | oral ingestion | 0.02 | 0.01 | Inta, 2014 |
|  | *Coleus amboinicus* Lour. | whole plants | rub with lime water | topical treatment | 0.02 | 0.01 | Chuakul et al., 2006 |
|  | *Premna herbacea* Roxb. | leaf, root, stem | concoction, decoction | bath, oral ingestion, poultice, sauna | 0.04 | 0.02 | Inta et al., 2011; Inta et al., 2013 |
|  | *Premna repens* H.R.Fletcher | leaf | grind | oral ingestion | 0.02 | 0.01 | Purintavaragul et al., 2012 |
|  | *Rotheca serrata* (L.) Steane & Mabb. | flower, leaf, root, stem | burn, decoction | oral ingestion, topical treatment | 0.08 | 0.04 | Chuakul, 2005; Bunsongthae and Chaiwong, 2010; Hutasingha, 2015; Maneenoon et al., 2015 |
|  | *Tectona grandis* L.f. | fruit, leaf, root, stem, wood | decoction | bath, oral ingestion, poultice, sauna | 0.08 | 0.04 | Tovaranonte, 2003; Pongamornkul, 2009; Inta et al., 2011; Inta et al., 2013 |
|  | *Vitex limonifolia* Wall. ex C.B.Clarke | stem | decoction | oral ingestion | 0.02 | 0.01 | Jeanwitchayakul, 2014 |
|  | *Vitex trifolia* L. | root, stem | decoction | bath, oral ingestion | 0.06 | 0.03 | Pongamornkul, 2009; Inta et al., 2011; Inta et al., 2013 |
| Lauraceae | *Cassytha filiformis* L. | whole plants | concoction | oral ingestion | 0.02 | 0.01 | Sukkho, 2008 |
| Leeaceae | *Leea indica* (Burm.f.) Merr. | stem, whole plants | concoction, decoction | bath, oral ingestion | 0.08 | 0.04 | Sukkho, 2008; Kamwong, 2009; Pongamornkul and Muangyen, 2012; Kadchumsang et al., 2015 |
|  | *Leea macrophylla* Roxb. ex Hornem. | root, stem | decoction | oral ingestion | 0.02 | 0.01 | Phongloy, 2015 |
|  | *Leea rubra* Blume | stem | concoction | oral ingestion | 0.02 | 0.01 | Kadchumsang et al., 2015 |
| Lythraceae | *Punica granatum* L. | leaf, stem | dried and concoction, grind | oral ingestion, topical treatment | 0.04 | 0.02 | Pongamornkul and Muangyen, 2012; Sumridpiem, 2017 |
| Malvaceae | *Abutilon hirtum* (Lam.) Sweet | leaf | decoction | oral ingestion | 0.02 | 0.01 | Neamsuvan et al., 2015 |
| Martyniaceae | *Martynia annua* L. | seed | decoction | oral ingestion | 0.02 | 0.01 | Sukkho, 2008 |
| Melanthiaceae | *Paris polyphylla* Sm. | rhizome | alcohol infusion | oral ingestion | 0.02 | 0.01 | Sukkho, 2008 |
| Melastomataceae | *Melastoma malabathricum* L subsp. *normale* (D.Don) Karst.Mey. | root, stem | decoction | not specified, oral ingestion | 0.06 | 0.03 | Srisanga, 1993; Winjchiyanan, 1995; Trisonthi et al., 2007 |
| Meliaceae | *Aphanamixis polystachya* (Wall.) R.Parker | not specified | not specified | not specified | 0.02 | 0.01 | Pongamornkul, 2009 |
|  | *Azadirachta indica* A.Juss. | not specified | not specified | not specified | 0.02 | 0.01 | Junsongduang et al., 2018 |
| Menispermaceae | *Stephania venosa* (Blume) Spreng. | stem | grind and pill | oral ingestion | 0.02 | 0.01 | Sumridpiem, 2017 |
|  | *Tiliacora triandra* (Colebr.) Diels | root | decoction | oral ingestion | 0.02 | 0.01 | Leeratiwong et al., 2016 |
|  | *Tinospora crispa* (L.) Hook.f. & Thomson | leaf, stem | decoction, none | bath, oral ingestion, poultice, sauna, topical treatment | 0.04 | 0.02 | Inta et al., 2011; Inta et al., 2013 |
| Moringaceae | *Moringa oleifera* Lam. | stem | dried and concoction | oral ingestion | 0.02 | 0.01 | Sumridpiem, 2017 |
| Musaceae | *Ensete glaucum* (Roxb.) Cheesman | ripe fruit | none | oral ingestion | 0.02 | 0.01 | Inta et al., 2011 |
|  | *Musa × paradisiaca* L. | fruit, ripe fruit | none | bath, oral ingestion, poultice, sauna | 0.04 | 0.02 | Inta et al., 2011; Inta et al., 2013 |
| Myristicaceae | *Myristica fragrans* Houtt. | seed | burn and pill | oral ingestion | 0.02 | 0.01 | Sumridpiem, 2017 |
| Myrtaceae | *Psidium guajava* L. | leaf | not specified | not specified | 0.02 | 0.01 | Pongamornkul, 2009 |
|  | *Syzygium aromaticum* (L.) Merr. & L.M.Perry | flower | grind and pill | oral ingestion | 0.02 | 0.01 | Sumridpiem, 2017 |
|  | *Syzygium cumini* (L.) Skeels | leaf, stem | decoction | oral ingestion | 0.02 | 0.01 | Phongloy, 2015 |
| Nelumbonaceae | *Nelumbo nucifera* Gaertn. | flower | burn and pill | oral ingestion | 0.02 | 0.01 | Sumridpiem, 2017 |
| Nymphaeaceae | *Nymphaea lotus* L. | flower, leaf | concoction | oral ingestion | 0.02 | 0.01 | Winjchiyanan, 1995 |
| Onagraceae | *Ludwigia hyssopifolia* (G.Don) Exell | root | decoction | oral ingestion | 0.02 | 0.01 | Yaso, 1997 |
| Oxalidaceae | *Averrhoa carambola* L. | root | concoction | oral ingestion | 0.02 | 0.01 | Sumridpiem, 2017 |
|  | *Biophytum umbraculum* Welw. | whole plants | decoction | topical treatment | 0.02 | 0.01 | Sukkho, 2008 |
| Peraceae | *Chaetocarpus castanicarpus* (Roxb.) Thwaites | leaf, young leaf | decoction | oral ingestion | 0.04 | 0.02 | Chuakul et al., 2004; Chuakul, 2005 |
| Phyllanthaceae | *Antidesma acidum* Retz. | stem | not specified | not specified | 0.02 | 0.01 | Pongamornkul, 2009 |
|  | *Phyllanthus amarus* Schumach. & Thonn. | leaf, stem, whole plants | concoction, decoction | oral ingestion | 0.06 | 0.03 | Inta et al., 2011; Inta et al., 2013; Sinworn and Viriyawattana, 2014 |
| Piperaceae | *Piper nigrum* L. | fruit | grind and pill | oral ingestion | 0.02 | 0.01 | Sumridpiem, 2017 |
|  | *Piper retrofractum* Vahl | fruit, inflorescences | decoction, grind and pill | oral ingestion | 0.04 | 0.02 | Inta et al., 2011; Sumridpiem, 2017 |
| Plantaginaceae | *Picrorhiza kurroa* Royle ex Benth. | rhizome | fresh and pill | oral ingestion | 0.02 | 0.01 | Sumridpiem, 2017 |
| Plumbaginaceae | *Plumbago indica* L. | root, stem | concoction, decoction, grind and pill, pounding | oral ingestion, poultice | 0.08 | 0.04 | Inta et al., 2011; Inta et al., 2013; Muangyen, 2013; Sumridpiem, 2017 |
| Poaceae | *Coix lacryma-jobi* L. var. *stenocarpa* Oliv. | root | decoction | oral ingestion | 0.02 | 0.01 | Nguanchoo, 2014 |
|  | *Oryza sativa* L. | seed | concoction | oral ingestion | 0.02 | 0.01 | Inta et al., 2011 |
|  | *Saccharum officinarum* L. | stem | concoction, decoction | oral ingestion | 0.04 | 0.02 | Inta et al., 2011; Sumridpiem, 2017 |
|  | *Zea mays* L. | corn cob, fruit | concoction, decoction | bath, oral ingestion, poultice, sauna | 0.04 | 0.02 | Inta et al., 2011; Inta et al., 2013 |
| Polygonaceae | *Bistorta officinalis* subsp. *officinalis* | root | grind and pill | oral ingestion | 0.02 | 0.01 | Sumridpiem, 2017 |
| Primulaceae | *Embelia ribes* Burm.f. | root, stem | decoction | oral ingestion | 0.02 | 0.01 | Chuakul, 2005 |
| Rhamnaceae | *Ventilago denticulata* Willd. | stem | concoction, decoction | oral ingestion | 0.06 | 0.03 | Pongamornkul, 2009; Inta et al., 2012; Kadchumsang et al., 2015 |
|  | *Ziziphus cambodiana* Pierre | stem | concoction, decoction | oral ingestion | 0.04 | 0.02 | Kamwong, 2009; Kadchumsang et al., 2015 |
|  | *Ziziphus mauritiana* Lam. | stem | concoction | oral ingestion | 0.02 | 0.01 | Kadchumsang et al., 2015 |
|  | *Ziziphus oenopolia* (L.) Mill. | stem | concoction | oral ingestion | 0.04 | 0.02 | Kamwong, 2009; Kadchumsang et al., 2015 |
| Rosaceae | *Rubus alceifolius* Poir. | root | not specified | not specified | 0.02 | 0.01 | Srisanga, 1993 |
|  | *Rubus ellipticus* Sm. | root, stem | decoction | oral ingestion | 0.02 | 0.01 | Trisonthi and Trisonthi, 2011 |
|  | *Rubus pirifolius* Sm. | stem | not specified | not specified | 0.02 | 0.01 | Trisonthi et al., 2007 |
|  | *Rubus rosifolius* var. *rosifolius* | not specified | not specified | not specified | 0.02 | 0.01 | Srisanga, 1993 |
|  | *Rubus sumatranus* Miq. | root | concoction | oral ingestion | 0.02 | 0.01 | Tangtragoon, 1998 |
| Rubiaceae | *Gynochthodes umbellata* (L.) Razafim. & B.Bremer | leaf | decoction | oral ingestion | 0.02 | 0.01 | Leeratiwong et al., 2016 |
|  | *Hymenodictyon orixense* (Roxb.) Mabb. | leaf, whole plants | cold water infusion, decoction, grind | bath, oral ingestion | 0.04 | 0.02 | Pongamornkul and Muangyen, 2012; Srithi, 2012 |
|  | *Ixora finlaysoniana* Wall. ex G.Don | stem | grind | not specified | 0.02 | 0.01 | Phatlamphu et al., 2021 |
|  | *Ixora grandifolia* Zoll. & Moritzi | stem | concoction | oral ingestion | 0.02 | 0.01 | Muangyen, 2013 |
|  | *Morinda citrifolia* L. | stem | dried and concoction | oral ingestion | 0.02 | 0.01 | Sumridpiem, 2017 |
|  | *Paederia linearis* Hook.f. | whole plants | decoction, fresh, grind and burn | bath, oral ingestion | 0.04 | 0.02 | Bunsongthae and Chaiwong, 2010; Phongloy, 2015 |
|  | *Paederia pilifera* Hook.f. | leaf | not specified | not specified | 0.02 | 0.01 | Pongamornkul, 2010 |
|  | *Pavetta tomentosa* Roxb. ex Sm. | stem | not specified | not specified | 0.02 | 0.01 | Pongamornkul, 2009 |
| Rutaceae | *Aegle marmelos* (L.) Corrêa | bark, fruit | concoction, decoction, pill | oral ingestion | 0.08 | 0.04 | Inta, 2008; Inta et al., 2011; Pongamornkul and Muangyen, 2012; Inta et al., 2013 |
|  | *Citrus × aurantiifolia* (Christm.) Swingle | stem | not specified | not specified | 0.02 | 0.01 | Pongamornkul, 2009 |
|  | *Melicope pteleifolia* (Champ. ex Benth.) T.G.Hartley | root | not specified | not specified | 0.02 | 0.01 | Trisonthi et al., 2007 |
|  | *Micromelum minutum* (G.Forst.) Wight & Arn. | root | not specified | not specified | 0.02 | 0.01 | Pipitkul, 2001 |
| Salicaceae | *Casearia grewiifolia* Vent. var. *grewiifolia* | bark, leaf | decoction | oral ingestion, sauna | 0.04 | 0.02 | Inta et al., 2011; Inta et al., 2013 |
| Sapindaceae | *Sapindus rarak* DC. | fruit | pill | oral ingestion | 0.02 | 0.01 | Inta, 2008 |
|  | *Schleichera oleosa* (Lour.) Oken | stem | concoction | oral ingestion | 0.02 | 0.01 | Kadchumsang et al., 2015 |
| Saururaceae | *Houttuynia cordata* Thunb. | leaf | none | oral ingestion | 0.02 | 0.01 | Sumridpiem, 2017 |
| Simaroubaceae | *Eurycoma longifolia* Jack | whole plants | not specified | not specified | 0.02 | 0.01 | Purintavaragul et al., 2012 |
| Smilacaceae | *Smilax blumei* A.DC. | stem | not specified | not specified | 0.02 | 0.01 | Leeratiwong et al., 2016 |
|  | *Smilax ovalifolia* Roxb. ex D.Don | root | not specified | not specified | 0.02 | 0.01 | Pongamornkul, 2009 |
| Solanaceae | *Solanum erianthum* D.Don | root | decoction | oral ingestion | 0.02 | 0.01 | Yaso, 1997 |
| Symplocaceae | *Symplocos racemosa* Roxb. | leaf, root, stem | decoction | oral ingestion, topical treatment | 0.02 | 0.01 | Sukkho, 2008 |
| Ulmaceae | *Holoptelea integrifolia* (Roxb.) Planch. | stem | concoction | not specified, oral ingestion | 0.04 | 0.02 | Pongamornkul, 2009; Kadchumsang et al., 2015 |
| Verbenaceae | *Lantana camara* L. | fruit | decoction | oral ingestion | 0.02 | 0.01 | Chuakul et al., 2006 |
|  | *Stachytarpheta jamaicensis* (L.) Vahl | whole plants | pill | oral ingestion | 0.02 | 0.01 | Inta, 2008 |
| Vitaceae | *Cissus quadrangularis* L. | stem, vine, wood | capsule, cooked with egg, decoction, eaten fresh, grind, hot water soak, pill, pulped, squash with water | oral ingestion, topical treatment | 0.25 | 0.1 | Trisonthi et al., 2007; Bunsongthae and Chaiwong, 2010; Sonsupub, 2010; Chotchoungchatchai et al., 2012; Purintavaragul et al., 2012; Srithi, 2012; Nguanchoo, 2014; Maneenoon et al., 2015; Panyadee, 2017; Sumridpiem, 2017 |
|  | *Leea guineensis* G.Don | root, stem | concoction, decoction | bath, oral ingestion, poultice, sauna | 0.04 | 0.02 | Inta et al., 2011; Inta et al., 2013 |
|  | *Tetrastigma mutabile* (Blume) Planch. | root, stem | decoction | oral ingestion | 0.02 | 0.01 | Chuakul, 2005 |
| Zingiberaceae | *Kaempferia parviflora* Wall. ex Baker | rhizome | decoction | oral ingestion | 0.02 | 0.01 | Srithi, 2012 |
