# Supplementary material for: An ethnobotanical review of medicinal plants used for treating hemorrhoids in Thailand
Source: Front Pharmacol. 2026 Apr 24;17:1705134. doi: 10.3389/fphar.2026.1705134 (PMC13152847; doi:10.3389/fphar.2026.1705134)
Supplement: Supplementary file 3 [file Table3.docx]

**Supplementary Table 3** Pharmacological activity review of the most important medicinal plants used to treat hemorrhoid in Thailand.

| **Scientific name** | **Family** | **Pharmacological activity** | **References** |
| --- | --- | --- | --- |
| *Aegle marmelos* (L.) Corrêa | Rutaceae | anti-inflammatory | Rajaram et al., 2018 |
|  |  | antioxidative activity | Reshma and Brindha, 2014 |
|  |  | antipyretic activity | Arul et al., 2005 |
|  |  | analgesic activity | Arul et al., 2005 |
| *Amaranthus spinosus* L. | Amaranthaceae | antidiarrheal activity | Hussain et al., 2009 |
|  |  | anti-inflammatory activity | Olajide et al., 2004 |
|  |  | analgesic activity | Paswan et al., 2020 |
|  |  | antioxidative activity | Sarker and Oba, 2019 |
| *Biancaea sappan* (L.) Tod. | Fabaceae | antioxidative activity | Suwan et al., 2018 |
|  |  | anti-inflammatory activity | Tewtrakul et al., 2015 |
|  |  | Vasorelaxant activity | Yan et al., 2015 |
|  |  | anti-bacterial activity | Vij et al., 2023 |
| *Cassia fistula* L. | Fabaceae | anti-inflammatory activity | Antonisamy et al., 2019 |
|  |  | antioxidative activity | Ilavarasan et al., 2005 |
|  |  | antipyretic activity | Gobianand et al., 2010 |
|  |  | antibacterial activity | Panda et al., 2011 |
| *Cissus quadrangularis* L. | Vitaceae | antioxidative activity | Chidambara Murthy et al., 2003 |
|  |  | anti-hemorrhoid activity | Pirshahid et al., 2016 |
|  |  | analgesic activity | Panthong et al., 2007 |
|  |  | anti-inflammatory activity | Panthong et al., 2007; Vijay and Vijayvergia, 2010 |
| *Croton persimilis* Müll.Arg. | Euphorbiaceae | antioxidative activity | Rattanapunya et al., 2021 |
| *Euphorbia tirucalli* L. | Euphorbiaceae | antioxidative activity | Le et al., 2021 |
|  |  | anti-inflammatory activity | Benitha et al., 2023 |
|  |  | antimicrobial activity | de Lima et al., 2021 |
| *Leea indica* (Burm.f.) Merr. | Leeaceae | antioxidative activity | Wong et al., 2012 |
|  |  | analgesic activity | Emran et al., 2012 |
|  |  | anti-inflammatory activity | Sakib et al., 2021 |
|  |  | wound healing activity | Azizi et al., 2016 |
| *Melastoma malabathricum* L subsp. *normale* (D.Don) Karst.Mey. | Melastomataceae | anti-inflammatory | Halim et al., 2022 |
|  |  | antioxidative activity | Halim et al., 2022 |
|  |  | wound healing | Halim et al., 2022 |
| *Millingtonia hortensis* L.f.. | Bignoniaceae | anti-inflammatory | Deethae et al., 2023 |
|  |  | antioxidative activity | Sivaraj et al., 2019 |
|  |  | antibacterial activity | Chiyasit, 2009 |
| *Mimosa pudica* L. | Fabaceae | anti-inflammatory activity | Patel and Bhutani, 2014b |
|  |  | antioxidative activity | Zhang et al., 2011; Lakshmibai and Amirtham, 2018 |
|  |  | wound healing | Kokane et al., 2009 |
|  |  | analgesic activity | Patro et al., 2015 |
| *Phyllanthus amarus* Schumach. & Thonn. | Phyllanthaceae | anti-inflammatory | Adedapo and Ofuegbe, 2015 |
|  |  | antinociceptive activity | Adedapo and Ofuegbe, 2015 |
|  |  | analgesic activity | Chandrashekar et al., 2005 |
|  |  | antioxidative activity | Lim and Murtijaya, 2007 |
| *Pluchea indica* (L.) Less. | Asteraceae | anti-inflammatory | Srisook et al., 2021 |
|  |  | antioxidative activity | Srisook et al., 2021 |
|  |  | antibacterial activity | Srimoon and Ngiewthaisong, 2015 |
|  |  | wound healing activity | Chiangnoon et al., 2022 |
| *Plumbago indica* L. | Plumbaginaceae | anti-inflammatory | Ittiyavirah et al., 2012 |
|  |  | antibacterial activity | Manyaem et al., 2022 |
|  |  | wound healing | Manyaem et al., 2022 |
|  |  | antioxidative activity | Eldhose et al., 2013 |
| *Rotheca serrata* (L.) Steane & Mabb. | Lamiaceae | antioxidative activity | Shashiraj et al., 2023 |
|  |  | antibacterial activity | Vidya et al., 2007 |
|  |  | anti-inflammatory | Vidya et al., 2007; Patel et al., 2014 |
| *Senna siamea* (Lam.) H.S.Irwin & Barneby | Fabaceae | anti-inflammatory activity | Lewis and Levy, 2011; Sagnia et al., 2014; Wadkhien et al., 2018 |
|  |  | antioxidative activity | Sagnia et al., 2014 |
| *Senna siamea* (Lam.) H.S.Irwin & Barneby | Fabaceae | analgesic activity | Ntandou et al., 2010; GF et al., 2018 |
|  |  | anti-inflammatory activity | Ntandou et al., 2010 |
|  |  | analgesic activity | Ntandou et al., 2010 |
|  |  | vasorelaxant activity | Busarakumtragul et al., 2010 |
| *Tadehagi triquetrum* (L.) H.Ohashi | Fabaceae | antioxidative activity | Song et al., 2023 |
|  |  | anti- inflammatory activity | Kalyani et al., 2011; Hermawati et al., 2020 |
| *Tectona grandis* L.f. | Lamiaceae | analgesic activity | Giri and Varma, 2015 |
|  |  | anti-inflammatory activity | Giri and Varma, 2015; Han et al., 2023 |
|  |  | antibacterial activity | Krishna and Jayakumaran, 2010 |
|  |  | wound healing | Varma and Giri, 2013a |
|  |  | antioxidative activity | Daramola, 2022; Han et al., 2023 |
|  |  | antimicrobial activity | Daramola, 2022 |
| *Thunbergia laurifolia* Lindl. | Acanthaceae | anti-inflammatory activity | Nanna et al., 2017 |
|  |  | analgesic activity | Nanna et al., 2017 |
|  |  | antioxidative activity | Essiedu et al., 2023 |
| *Ventilago denticulata* Willd. | Rhamnaceae | antioxidative activity | Pongjanta et al., 2016; Srimoon et al., 2020 |
|  |  | anti-inflammation activity | Sornprasert et al., 2012 |
| *Vitex trifolia* L. | Lamiaceae | anti-inflammatory activity | Ghafari et al., 2021 |
